# Functionalized 12 µm Polyethylene Separator to Realize Dendrite‐Free Lithium Deposition toward Highly Stable Lithium‐Metal Batteries

**DOI:** 10.1002/advs.202102215

**Published:** 2022-03-07

**Authors:** Qiannan Zhao, Ronghua Wang, Xiaolin Hu, Yumei Wang, Guanjie Lu, Zuguang Yang, Qiwen Liu, Xiukang Yang, Fusheng Pan, Chaohe Xu

**Affiliations:** ^1^ College of Aerospace Engineering Chongqing University Chongqing 400044 P. R. China; ^2^ College of Materials Science and Engineering Chongqing University Chongqing 400044 P. R. China; ^3^ National University of Singapore (Chongqing) Research Institute Chongqing 401123 P. R. China; ^4^ Hunan Province Key Laboratory of Electrochemical Energy Storage and Conversion School of Chemistry Xiangtan University Xiangtan 411105 P. R. China; ^5^ National Engineering Research Center for Magnesium Alloys Chongqing University Chongqing 400044 P. R. China

**Keywords:** composite separator, dendrite‐free deposition, functionalization, lithium anode, lithium metal batteries

## Abstract

Direct application of metallic lithium (Li) as the anode in rechargeable lithium metal batteries (LMBs) is still hindered by some annoying issues such as lithium dendrites formation, low Coulombic efficiency, and safety concerns arising therefrom. Herein, an advanced composite separator is prepared by facilely blade coating lightweight and thin functional layers on commercial 12 µm polyethylene separator to stabilize the Li anode. The composite separator simultaneously improves the Li ion transport and lithium deposition behaviors with uniform lithium ion distribution properties, enabling the dendrite‐free Li deposition. As a result, the lithium anode can stably cycle up to 3000 cycles with the high capacity of 3.5 mAh cm^−2^. Moreover, the composite separator exhibits wide compatibility in LMBs (Li–S and Li‐ion battery) and delivers stable cycling performance and high Coulombic efficiency both in coin and lab‐level soft‐pack cells. Thus, this cost‐effective modification strategy exhibits great application potential in high‐energy LMBs.

## Graphical abstract

The designed functionalized layer can regulate lithium (Li) ions distribution and ensure uniform Li ions flux and deposition even in the presence of rough defects or cracks disturbance. Therefore, ultralong cycling dendrite‐free Li anodes can be achieved with this special designed TV‐PE separator, endowing great potential in the practical application of future high energy lithium metal batteries.

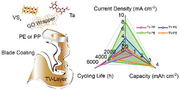

## 1 Introduction

Rechargeable lithium metal‐based batteries (LMBs) have refueled research and industry community's interest due to their potential application in personal electronics, electric vehicles and aerospace fields.^[^ ^1^ ^]^ By replacing conventional graphite anode (372 mAh g^−1^) with metallic lithium (Li), the energy density at a cell level is expected to increase by 40–50% because of its high theoretical capacity (3860 mAh g^−1^) and the lowest electrochemical potential (−3.04 V vs the standard hydrogen electrode).^[^ ^2^ ^]^ Although Li is the ideal candidate for high energy density storage systems, its practical application is still hindered by unstable Li ion stripping and deposition, formation of Li dendrites and “dead lithium,” and potential safety issues. Specifically, the surface of lithium anode inevitably has microscopic roughness that will cause the concentration gradient of the Li ions. Li ions will preferentially deposit on the protuberant tips with a self‐enhanced effect, and finally cause the formation of Li dendrites and “dead lithium.” Additionally, the exposed Li dendrites are further consumed with the electrolyte, leading to large charge transfer resistance and low Coulombic efficiency.^[^ ^3^ ^]^ Therefore, how to promote the Li ion transportation and lower the overpotential is critical for a dendrite‐free Li anode. In this context, various strategies such as developing Li‐metal alloy (Li‐Au ^[^ ^4^ ^]^, Li‐Mg ^[^ ^5^ ^]^ etc.), designing porous current collectors ^[^ ^6^ ^]^ and constructing the artificial solid electrolyte interface (SEI) layer ^[^ ^7^ ^]^ have been employed to solve the dendrites problem. On the other hand, optimization of the electrolyte to produce a stable SEI and using solid state electrolyte are also considered as efficient ways to stabilize and improve the electrochemical performance of Li anode.^[^ ^8^ ^]^ Unfortunately, these strategies usually involve tedious and dangerous manufacturing process because of the high chemical activity nature of lithium, which is not economical and industrially viable for large scale production.^[^ ^9^ ^]^

In addition to the direct modification of lithium anode and electrolyte optimization, it is noteworthy that modification of separators is also a promising strategy to regulate the lithium ions deposition/stripping behavior and inhibit the lithium dendrites formation due to their direct contact with the lithium anode. Some ceramic coating materials such as Al_2_O_3_,^[^ ^10^ ^]^ Mg(OH)_2_,^[^ ^11^ ^]^ and LLZTO^[^ ^12^ ^]^ have been developed and employed as functional layer of separators in the published works. Although these ceramics coating could mechanically inhibit the dendrites formation, the coating layers are inevitably thick and heavy because of their high density with relatively large interface resistance. Given that the important parameters, such as thickness and mass loading of the functional layer have a great influence on the energy density from the overall perspective of the LMBs system, graphene, and other 2D materials with the advantages of light weight, large specific surface area, and good mechanical robustness are considered as ideal coatings for suppressing lithium dendrites.^[^ ^13^ ^]^ However, graphene coating alone is difficult to regulate the lithium deposition and stripping and ensure extra‐long lithium cycling. Additionally, some preparation methods for graphene film such as vacuum filtration is not suitable for large‐scale production in industrial applications.^[^ ^14^ ^]^ Therefore, how to construct a lightweight composite separator that can efficiently regulate the lithium deposition/stripping behavior through a simple and scalable manufacturing process is still a big challenge for high‐energy lithium metal batteries.

Herein, we have designed and prepared an advanced composite separator with total thickness of 15 µm by coating the polyethylene separator with a functional layer (TV layer) containing reduced graphene oxide (rGO), tannic acid (TA), and VS_4_ (named as TV‐PE separator). Compared with the original PE separator, the TV‐layer regulates high Li^+^ transport with improved lithium transference number from 0.48 to 0.68. Furthermore, combined with the adjustment of the SEI, the Li ion fluxes are guided to achieve a uniform lithium deposition, and the compact cobblestone like lithium deposition is achieved with the TV‐PE separator even when the lithium deposition capacity is as high as 30 mAh cm^−2^. Experimental and simulation results have confirmed that TV‐PE separator can regulate the lithium to horizontally grow into dense cobblestone‐like lithium crystals when a disturbance happens rather than needle‐ or moss‐like lithium dendrites. As a result, the modified separator can well solve the concerns of lithium dendrites and microshort circuit amplification effect, enabling a dendrite‐free Li anode up to 6000 and 4200 h cycling for the 25 µm TV‐PP separator and 15 µm TV‐PE separator, respectively. All these positive effects ensure the ultralong cycling dendrite‐free Li anode with our specially designed separator and its wide practical application in promising LMBs.

## 2 Design of the Functional TV‐Layers on Commercial Separator

**Figure 1:**
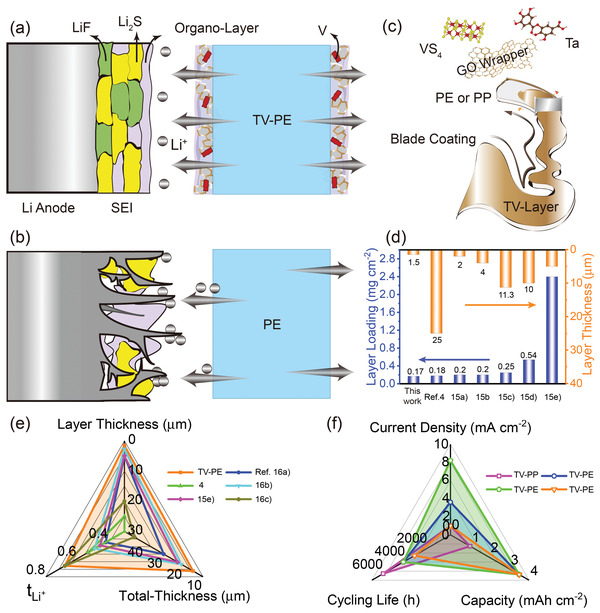
Cartoon drawing of the Li ion deposition on the Li anode with a) TV‐PE and b) PE separator. c) Preparation illustration of the TV‐PE separator. d) Comparison of the functional layer loading and loading thickness of the designed TV‐PE separator with other modified separator reported in literatures.^[^ ^4^, ^15^ ^]^ e) Comparison of the layer thickness, the total separator thickness, and the lithium ion migration number of the TV‐PE separator with other modified separators.^[^ ^4^, ^15^, ^16^ ^]^ f) Metal lithium anode deposition and stripping performance regulated by the TV‐PP and TV‐PE separator.

The design concept of functionalized separator is illustrated in **Figure** **1**a,b. As is known, uneven lithium ion distribution will induce the formation of Li ions concentration and migration gradient in the battery with unmodified polyolefin separator, and therefore lithium dendrites are easy to form and grow arbitrarily. Here, the multifunctional TV‐layer containing reduced graphene oxide (rGO), tannic acid (TA), and VS_4_ was specially designed and modified onto both sides of commercial polyolefin separators aiming to enable a uniform Li ion distribution and fast ion transport. Combined with the adjustment of the SEI structure, Li ion fluxes are guided by the designed TV‐polyolefin separator so that the lithium deposition behavior can be further regulated for dendrite‐free lithium anode. To facilitate the coating process and enhance the adhesion between the coating slurry and polyolefin matrix, the hydrophilicity of polyolefin film was first improved by preoxidation. The fourier transform infrared (FT‐IR) spectra reveal that C—O—C and —OH groups appeared on the treated membrane, indicating that the hydrophilic surface was successfully achieved (Figure S1, Supporting Information). These oxygen functional groups can improve the binding between the polyolefin matrix and the TV layer through chemically interacting with the GO wrapper and TA component and binding each other through hydrogen bond. Afterward, the slurry composed of TA, VS_4_, and rGO sheets was coated on the pretreated polyolefin matrix to act as functional layer (Figure 1c), in which TA is added in the functional layer to enhance the ionic conductivity of the composite separator, while VS_4_ is expected to modify the SEI structure on the lithium anode.^[^ ^17^ ^]^ 2D rGO sheets with large‐aspect‐ratio are chosen to improve the separator surface, also work as the binder to wrapped TA and VS_4_ (Figure S2a–e, Supporting Information).

The commercial PE membrane with thickness of 12 µm was chosen as the matrix because the ultrathin separator would be a key solution of high energy traction battery for future electric vehicle. For the advanced separator modification strategies, the mass loading and the thickness of the functional layer, the total thickness and the lithium ion migration number (*t*_Li_^+^) of the designed separator are closely related to the financial cost, energy density, and the cycling performance of the lithium metal the battery. Figure 1d–f compares the functional layer mass loading, thickness, and *t*_Li_^+^ of the designed separator with some other advanced separators. As observed in Figures S2g and S3a,b (Supporting Information), the thickness of the TV‐layer is about 1.5 µm; and the mass loading is as light as ≈0.15 mg cm^−2^ on each side, which is much lower than some advanced separator modification strategies (Figure 1d) and the commercial ceramic coated separator (3.0 µm and 0.45 mg cm^−2^ on each side). As the coating layer is thin, it has no influence on the electrical insulting property of the separator. The resistances of both the pristine PE and the designed TV‐PE separator are both out of the range when being tested on a four probes tester (Figure S3c,d, Supporting Information) which means both of them are electrically insulated. Not only that, the designed TV‐PE separator also achieve a high *t*_Li_^+^ of 0.68, which guarantee the long cycling performances of the lithium metal anode with high capacity of 3.5 mAh cm^–2^ with different current densities (Figure 1e,f).

**Figure 2:**
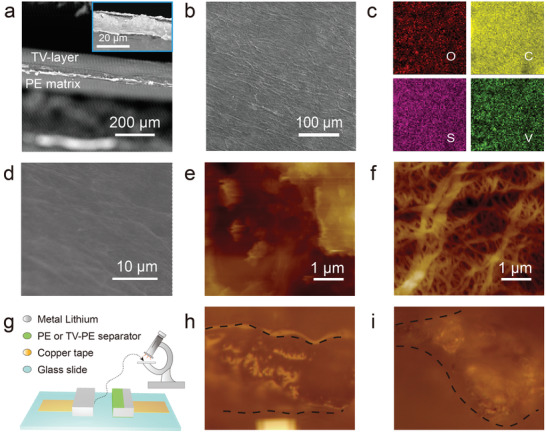
Micromophology characterization of the separators. Cross‐section a), top‐view SEM images and corresponding energy dispersive X‐ray spectroscopy (EDS) mappings b–d) of the as‐prepared TV‐PE separator. AFM images of e) the TV‐PE and f) the original PE separator. Optical observation of the lithium deposition behavior with different separators. g) Optical observation cell model for the lithium stripping and deposition. Optical images of the Li anode after 1.5 h deposition regulated by h) TV‐PE and i) PE separator.

The cross‐section scanning electron microscope (SEM) and atomic force microscopy (AFM) images in **Figure** **2**a; and Figure S2f,g (Supporting Information) show the compact sandwich structure of the TV‐PE film, while its surface is quite flat and well wrapped with the rGO sheets (Figure 2b–e), which is favorable for uniform electrolyte and ion flux distribution.^[^ ^18^ ^]^ The original PE membrane possesses a porous structure with uneven distribution of macrochannels between 100 and 200 nm (Figure 2f). It should be noted that after TV‐layer modification, the mass transport realizes mainly through the meso‐channels and microchannels formed in the functional layer, which could greatly facilitate the mass transport.

## 3 Advanced TV‐Polyolefin Separator for Dendrite‐Free Lithium Anode

### 3.1 The Lithium Deposition Performance Regulated by the Functional TV‐Layer

The lithium deposition processes with TV‐PE separator and original PE separator were first recorded by optical microscopy in a homemade cell model (Figure 2g). During deposition, as seen in Figure S4a–c (Supporting Information), the TV‐PE separator regulates an intact lithium deposition without dendrites even after 1.5 h (Figure 2h). But for the cell with a PE separator, obvious dendritic lithium formed after 0.5 h deposition (Figure S4d,e, Supporting Information) and gradually grew into large moss lump after 1.5 h deposition (Figure 2f). The dead dendritic lumps will finally piece the separator in a practical battery and inevitably induce safety issues. These results optically demonstrate the exceeding ability of the TV‐PE separator in regulating the lithium deposition and suppressing the dendrites growth.

**Figure 3:**
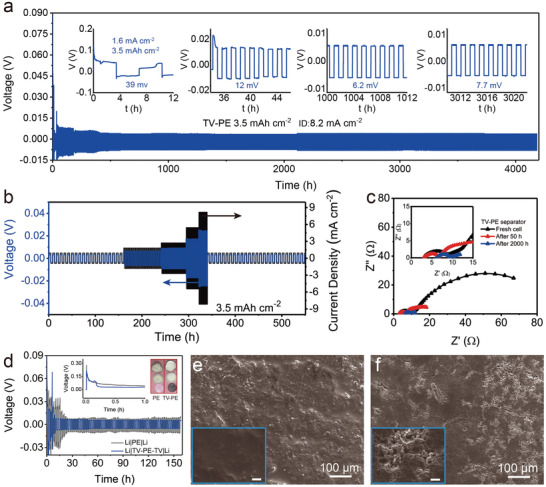
Electrochemical performance of Li–Li symmetric cells with the TV‐PE separator. The capacity is 3.5 mAh cm^−2^ in all batteries. a) Voltage profiles at high current densities of 8.2 mA cm^−2^. The battery was first activated at low current density of 1.6 mA cm^−2^ with the same capacity of 3.5 mAh cm^−2^ for 5 cycles. b) Rate performance of Li//TV‐PE//Li at current densities of 0.9, 1.8, 2.7, 5.5, and 8.2 mA cm^−2^. Then the current density went back to 0.9 mA cm^−2^. c) EIS results of Li//TV‐PE//Li cell before and after cycling. d) Voltage profiles of Li//PE//Li and Li//TV‐PE//Li symmetric batteries at a current density of 2.6 mA cm^−2^, inset: initial deposition profiles and corresponding photos of the disassembled batteries after cycling. SEM images of Li surface after 158 h cycling with e) TV‐PE separator and f) PE separator. The scale bar is 10 µm in the insets of e) and f). All stripping and deposition capacities were fixed at 3.5 mAh cm^−2^.

To get closer to practical industrial parameters, a plating/stripping area capacity of 3.5 mAh cm^−2^ was testified in the lithium symmetric battery. As the results shown in **Figure** **3**a; and Figure S6a (Supporting Information) Li//TV‐PE//Li batteries can stably cycle for several thousand hours. Even when the current density is as high as at 8.2 mA cm^−2^, the battery shows 4200 h cycling life (more than 3000 cycles). Furthermore, the Li//TV‐PE//Li symmetric battery shows a good rate performance as observed in Figure 3c, implying the high reliability in practical application. Electrochemical impedance spectroscopy (EIS) of Li//TV‐PE//Li symmetric battery displays a much more stable interfacial resistance than the Li//PE//Li symmetric battery during cycling (Figure S5a,b, Supporting Information). The values of the impedance parameters are summarized in the Table S1 (Supporting Information). By contrast, the ionic conductivity of the Li//TV‐PE//Li battery (1.8 × 10^−4^ S cm^−1^) is much higher than that of Li//PE//Li (1.5 × 10^−5^ S cm^−1^), while the *D*_Li_^+^ of the two batteries is of the same order of magnitude, illustrating that the Li ion transportation and kinetics are greatly enhanced after separator modification. Excitingly, the TV‐PE separator also shows an excellent compatibility in carbonate‐based electrolyte and ensures a good stabilizing effect on the Li anode for more than 3200 h continuous cycles at 1 mA cm^−2^ and 3.5 mAh cm^−2^ (Figure S6b, Supporting Information). In order to further evaluate lithium planting/stripping performances with different separators, half Cu–Li batteries using different separators were further tested (Figure S6c–f, Supporting Information). Clearly, Cu–Li batteries with designed TV‐PP and TV‐PE separator show much more stable lithium planting and stripping performances with Coulombic efficiency of more than 97% for the first 50 cycles. In contrast, the batteries with the pristine PP and PE separator present random voltage oscillation and much lower Coulombic efficiency during cycling. To sum up, the as‐fabricated TV‐polyolefin separators demonstrate a good compatibility in either ester or carbonate‐based electrolytes and stable planting/stripping performance for half Cu‐Li batteries with high CE so that they can be well used in practical LMBs systems.

The cycled Li–Li symmetric cells with PE and TV‐PE separators at different cycling stages were disassembled to analyze the function of separators. After the quick activation process which is related to the formation of SEI layer,^[^ ^19^ ^]^ the Li//TV‐PE//Li symmetric cell delivers a much lower and more stable voltage hysteresis than Li//PE//Li cell (Figure 3d). After 400 h cycling, the TV‐layer presents a uniform micromorphology and soft rGO sheets still tightly envelop the TV functional layer (Figure S7, Supporting Information), while the surface of Li anode coupled with TV‐PE separator is smooth and flat without any cracks as the cycling life goes on from 158 h (the inset in Figure 3d,e), 400 h (Figure S8c, Supporting Information) to even 4200 h (Figure S8e, Supporting Information). In the case of Li//PE//Li cell, however, although no short‐circuit happened for a short cycling life of 158 h cycling, the Li anode shows uneven SEI surface with visible cracks and a lot of whiskers (Figure 3f). As the cycle goes on, the surface will further deteriorate and the exposed Li dendrites will continuely react with the liquid electrolyte to form severe mossy mophology, which finally cause the sturcture pulverization (Figure S8b,d), Supporting Information.

The lithium deposition and stripping behavior with rGO‐PE, rGO/TA‐PE, rGO/VS_4_‐PE, and TV‐PE separator were further tested, as shown in Figure S9 (Supporting Information). EIS of lithium symmetric batteries with different separators before and after cycling further employed to distinguish the interfacial conditions (Figure S9a,b, Supporting Information). Clearly, the symmetric battery with TV‐PE separator displays the smallest charge transfer resistance compared with other counterparts before and after cycling, revealing the synergistic effect of rGO, TA, and VS_4_ in promoting reversible ion transport. Compared with the other three separators, TV‐PE separator also gives the battery lowest overpotential and most stable cycling performance (Figure S9c, Supporting Information) which suggest the most stable interface between the TV‐layer and the lithium surface. Moreover, the planting/striping curve of Li//TV‐PE//Li is quasirectangular, meaning that the functional layer with micrometer scale thickness between polyolefin and lithium surface can work as an artificial SEI layer.

The TV‐layer was also modified on another Celgard 2500 PP membrane to construct a TV‐PP composite separator. As depicted in Figure S10a (Supporting Information), the as‐constructed Li//TV‐PP//Li symmetric battery could cycle more than 6000 h with a small overpotential of 15.5 mV. This is the best performance of lithium plating and stripping as far as we know (compared with some published works Table S3, Supporting Information), clearly proving the distinctive function of the TV‐layer. Noteworthy, the overpotential tends to increase after the long‐term cycle indicating relatively lean lithium ion concentration with this long time cycling. Then the battery was disassembled to detect the internal changes. As seen in the inset of Figure S10c (Supporting Information), the Li anode still shows bright metallic appearance, and the surface was covered by dense cobblestone‐like deposition, indicating that the lithium growth behavior is well regulated rather than forming dendrites or pulverized lithium after this long time cycling. The symmetric battery with a TV‐PP separator also shows a stable cycling performance with stable overpotential when the current density and the deposition capacity increase to 8.2 mA cm^−2^ and 5.47 mAh cm^−2^ (Figure S10d, Supporting Information). The partial enlarged views in Figure S10e–i (Supporting Information) show that the deposition/stripping profiles was gradually stabilized into a decent rectangular‐like with ambiguous nucleation overpotential during cycling. So how does the TV‐layer ensure the gentle lithium deposition behavior?

### 3.2 The Function of the TV‐Layer in Regulating Lithium Deposition Behavior

**Figure 4:**
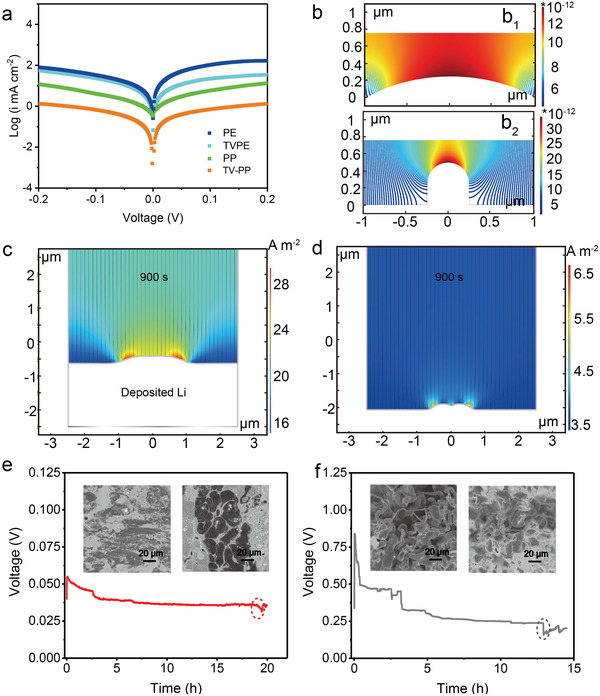
a) Tafel plots of the lithium symmetric batteries with different separators. Theoretical simulation of the inner SECD of the TV‐PE separator b_1_) and PE separator b_2_). Simulation of the relative Li deposition after 900 s with e) TV‐PE and f) PE separator. Voltage‐time profiles of lithium symmetric batteries regulated by e) TV‐PE, and f) PE separator. The insets show the corresponding micromorphologies of lithium deposition when disturbance happens as the red and gray marks in e) and f), respectively.

The exchange current density (i_0_) in a Li symmetrical battery reflects the kinetic resistance of the Li deposition/stripping process. High i_0_ usually indicates the fast charge transfer at the SEI/lithium interface in the battery.^[^ ^20^ ^]^ As shown in **Figure** **4**a, the value of i_0_ with the TV‐polyolefin and the original polyolefin (PE and PP) separators are calculated according to the Tafel profiles. i_0_ of TV‐PE and TV‐PP is 0.50 and 0.31 mA cm^−2^, respectively, much higher than that of PE (0.29 mA cm^−2^) and PP (0.15 mA cm^−2^, Table S2, Supporting Information), indicating that TV‐layer can lower the interfacial charge transfer barrier between Li anode and separator. As discussed above, TV‐layer modification clearly regulates the separator surface which may further impact the separator/Li anode interface. Hence, the surface electrolyte current densities (SECD) with TV‐PE and PE separators were simulated by COMSOL. The veil‐like and irregular macrogrids morphology of TV‐PE and pristine PE separators was modeled by different microsize curvatures respectively, as seen in Figure 4b. The TV‐PE model with larger curvature shows a relatively uniform SECD corresponding to the slow profile transition (Figure 4b_1_). Yet because of the irregular and sharper curvature change, the original PE separator displays wide SECD with higher current density concentrating on the top of the bulge (Figure 4b_2_). The SECD inside the battery represent the ion distribution and can directly affect the lithium deposition behavior. The uniform distribution of the SECD guaranteed by the TV‐layer can efficiently prohibit the formation of the space charge region at the Li anode/separator interface. However, the SECD concentrated area with the original PE separator will inevitably induce faster lithium deposition on the corresponding Li surface, resulting in dendrites or moss lithium formation. These results also echo that TV functional layer regulates higher lithium ion migration number (*t* ^+^), which remarkably increased from 0.30 and 0.48 for PP and PE separator to 0.59 and 0.68, respectively, after modification (Figure S11a–d and Table S2, Supporting Information).^[^ ^21^ ^]^ Additionally, *t* ^+^ of the rGO/TA‐PE, rGO/VS_4_‐PE, and rGO‐PE separator in the battery (Figure S11e–g, Supporting Information) was further tested to clarify the function of the rGO, TA, and VS_4_ in the TV layer. The *t* ^+^ values are 0.61 for rGO/TA‐PE, 0.55 for rGO/VS_4_‐PE, and 0.52 for rGO‐PE separator, which are smaller than that of the TV‐PE separator (0.68) but bigger than the pristine PE separator (0.48). Figure S11h (Supporting Information) shows the chemical composition analysis of the pristine PE, TA powder, cycled rGO‐PE, and cycled rGO/TA‐PE. During cycling, hydroxyl groups in TA on the surface of TV layer would interact with lithium to generate lithium‐alkoxides at the interface of TV‐layer and lithium. According to literatures, these kind lithium‐alkoxides could greatly decrease the interfacial resistance and reduced lithium deposition overpotential.^[^ ^17^, ^22^ ^]^ Therefore, introducing TA in the TV‐layer could greatly improve the lithium ion transportation. TA and VS_4_ wrapped by rGO sheets in the TV‐layer paly synergistic effects in improving the lithium ions transport ability and regulate the lithium deposition behavior together.

The Li deposition process was further simulated. As is known, the defect region on the surface of Li anode will also affect the distribution of lithium ions and induce preferential deposition of lithium ions onto the tiny protuberance. Thus, we introduced two bulges with 200 nm height in the simulation models to represent the roughness disturbance on the Li anode surface. The simulation model contains the separator and the Li anode with liquid electrolyte between them (Figure S12, Supporting Information). The Li ions conductivity with different separator (Table S1, Supporting Information) was chosen as the predominant parameter during simulation. It is found that the TV‐PE module displays a uniform Li^+^ flux and fast Li ion deposition at the corner region of the bulges, attenuating the tip nuclei and the corresponding amplification effect (Figure S13, Supporting Information). After 900 s deposition, 1.88 µm of lithium was deposited onto the flat region in the TV‐PE module which is more than twice height of the PE separator module (0.86 µm) (Figures S14 and S15, Supporting Information). As a result, the angular corner disappears in the TV‐PE module during the deposition process; however, the obvious curvature still exists in the PE module (Figure 4c,d). It can be concluded that the functional TV‐layer could regulate the Li ions distribution and ensure uniform Li ions flux distribution and deposition. Thus, even with the presence of certain defects or instable deposition disturbance, the TV‐PE separator could also avoid the sharp nuclei and the resultant dendrites amplification effect.

Long time deposition experiments further reveal the lithium deposition behavior. A slowly voltage drop appears in the voltage profile after 19 h operating in the Li//TV‐PE//Li symmetric cell (Figure 4e). After that, some cobblestone‐like lithium is observed in the disassembled Li anode (Figures S16a–h and S17, Supporting Information), which accords well with the lithium deposition morphology after 6000 h cycling with the TV‐PP separator in Figure S10c (Supporting Information). Because of the stress mitigation effect of the TV layer during cycling (Figure S18, Supporting Information), the voltage changes slightly and short circuit does not happen immediately, as seen in Figure 4e. According to the simulation, the TV‐layer can regulate a uniform ionic distribution and fast lithium ion deposition. When peculiar nucleation sites exist in the local scene, the Li/TV‐layer interface will provide a uniform ion supply to guide horizontal lithium growth and naturally form the cobblestone‐like lithium instead of sharp dendrites. Such cobblestone‐like lithium deposition is considered as an effective way to avoid the formation of Li dendrites and direct short‐circuit in the battery.^[^ ^23^ ^]^ On the contrary, the Li anode was fully covered by loose Li dendrites with a PE separator (Figure S16i–p, Supporting Information), causing the large vertical drop of the voltage and direct short circuit in Figure 4f.

The lithium deposition/striping behavior and the TV layer changes over time and deposition capacity were further recorded specifically. As seen in Figures S19 and S20 (Supporting Information), the TV layer shows maintain a smooth and uniform surface after depositing for 1, 3, 5, 8, and 10 h. As deposition time goes on, some visible cobblestone lithium appears after 15 h and gradually increase after 18 and 20 h. At the same time, these high capacity lithium deposition with huge stress at the TV layer‐lithium interface produce amplified cracks on the TV layer, and the continuous TV layer is broken into small fragments (after 20 h deposition). Impressively, the cross‐sectional images show that the deposited lithium remains compact even after 20 h deposition (deposition capacity up to 30 mAh cm^−2^) (Figure S21, Supporting Information). The dense cobblestone lithium morphology was also captured as seen in Figure S21k (Supporting Information). In sharp contrast, in the case of original PE separator, the lithium deposition represents obvious lithium whisker after only 3 h deposition and gradually grows into mossy dendrites after 5 and 8 h deposition (Figure S22, Supporting Information). These results confirm the efficient lithium transport regulation and high lithium deposition capability of the TV‐PE separator.

The mechanical performance of the TV‐layer was then tested by the peeling and indentation tests. Figure S23a,b (Supporting Information) shows that the TV‐layer has a moderate 180° binding strength of 0.38 kN m^–−1^. After peeling test, there is some TV‐layer still adheres to the PE matrix, indicating the good adhesion of the layer (Figure S23b,c, Supporting Information). For the indentation test, the TV layer exhibits higher indentation force at the same indentation and greater hardness with similar contact stiffness compared with PE separator (Figure S23d,e, Supporting Information). These results represent that the TV‐layer improve the mechanically robust of the separator, which is beneficial for alleviating the volume change during lithium depositing and stripping processes, especially for the high capacity lithium deposition. Although the TV‐layer after cycling shows lower Er than before cycling due to the formation of organic‐SEI components from electrolyte decomposition, the contact stiffness and hardness are still superior to that of the original PE membrane (Figure S23e, Supporting Information).

**Figure 5:**
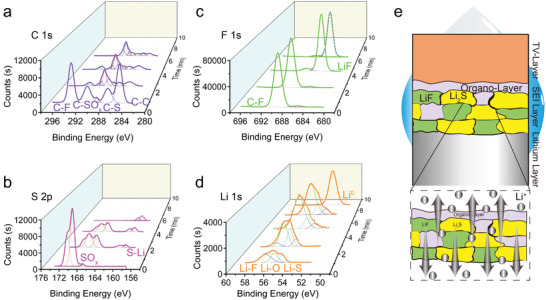
Composition and thickness qualitative analysis of SEI layer on the lithium anode protected by a TV‐PE separator. XPS spectra of a) C1s, b) F1s, c) S2p, and d) Li 1s along depth direction sputtered by Ar^+^ for every 2 min. e) Schematic diagram of spatial composition distribution between the TV‐layer and the Li electrode.

X‐ray photoelectron spectroscopy (XPS) was further used to detect the chemical environment change before and after cycling. As seen in Figure S24 (Supporting Information), compared with the original V 2p spectrum, obvious V^0^ peaks appeared at 514.1 and 521.5 eV after cycling which may come from the reduction of VS_4_ by lithium. At the same time, in‐depth XPS by Ar^+^ sputtering reveals that the SEI composition on the Li anode is regulated by TV layer. As seen in **Figure** **5**a–d, the C1s spectra reveal that the top layer of the SEI is composed of C—C, C—SO*_x_*, C—S, and C—F bonds, and the peak strength decreases along the depth direction; F1s spectra display that the bonds change from C—F on surface to the inner LiF; for the S2p depth profiling, the surface SO*_x_* gradually transform into Li_2_S as the Ar^+^ etching going; and Li1s environments change from a mixture of Li—F, Li—S, and Li—O bonds to single Li(0), which agrees well with the F1s, S2p, and the FT‐IR spectra (Figure S11h, Supporting Information). Based on these composition detection results along depth direction, it is revealed that SEI contains a thin organo‐layer on the surface and a mixed phase of LiF and Li_2_S in the interior. The organo‐layer formed by electrolyte deposition is soft and beneficial for buffing the volume expansion of lithium. The inner layer of inorganic LiF and Li_2_S mixture can prevent electrons leakage and improve the ionic conductivity of SEI layer.^[^ ^24^ ^]^ Such ideal SEI layer can efficiently reserve the electrolyte well and inhibit the dendrites formation during cycling, as schemed in Figure 5e. Additionally, EDS mappings of the cycled TV layer and lithium anode further identify the modified SEI components. For the cycled TV‐layer, element C, O, F, N, V distribution fit well with the TV layer outline, while the element S distribution is blurry. At the same time, strong and uniform element S signal appears on the cycled lithium electrode surface which could attribute to the top surface organo‐sulfur species and the deep layer Li_2_S in the SEI originating from the VS_4_ in the TV layer.

To conclude, in the designed TV layer, TA can enhance the ionic conductivity of the battery and together with VS_4_ to modify the SEI structure on the lithium anode, while veil‐like rGO sheets could both improve the separator surface and work as the binder to wrapped TA and VS_4_, achieving a win‐win effect of the three. These three components play a synergistic function in regulating lithium interfacial electrochemistry to ensure the dendrite‐free lithium deposition.

## 4 Electrochemical Performance of Typical LMBs with TV‐PE Separator

**Figure 6:**
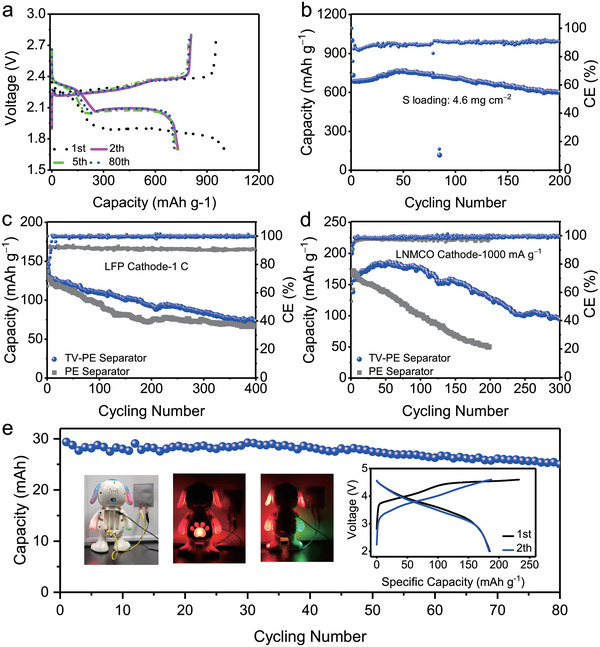
Electrochemical performances of LSBs with TV‐PE separator. a) Discharge and charge profiles at different cycles at 0.2 C, b) cycling performance and the corresponding Coulombic efficiencies (CE). Evaluation of the electrochemical performances with different separators and Li metal anode in LMBs. c) Cycling performances of commercial LFP cathode at 1 C rate. d) Cycling performances of lithium‐rich‐layered oxide (LMNC‐622) cathode. e) Evaluation of the cycling performances of soft‐packaged thin battery with Li_1.4_Mn_0.6_Ni_0.2_Co_0.2_O_2.4_ (LMNC‐622) cathode. The total mass of the active material is 159 mg in the cathode with area loading of 5.3 mg cm^−2^.

LSBs with high sulfur loading (4.6 mg cm^−2^) cathode were assembled to evaluate the function of the designed TV‐PE separator. Because of the high sulfur loading, the first cycle involves the redistribution process of active materials and the stabilization of the electrode interface (**Figure** **6**a). After the first cycle, impressively, high specific capacities of 1044.8 and 999.8 mAh g^−1^ (equaled to 4.8 and 4.6 mAh cm^−2^) are delivered at 0.1 C (Figure S26, Supporting Information). Moreover, the battery with TV‐PE separator showed much higher Coulombic efficiencies than that with PE separator, which was mainly attributed to the fast Li ions transportation and stable Li ions deposition effect of the TV‐layer. The cycling performance of LSB with TV‐PE separator is exhibited in Figure 6b, and more than 3 mAh cm^−2^ was remained after 150 cycles at 0.2 C. Considering the high sulfur loading of the cathode, the electrochemical performance can be further improved by changing the charging‐discharging manner, i.e., discharging at low current density and charging at high current density or properly lowering the sulfur loading (Figure S27, Supporting Information). On the other hand, XPS results (Figure S28, Supporting Information) and the optical adsorption experiment (Video S1, Supporting Information) reveal the efficient role of the TV‐layer in adsorbing polysulfides. After interacting with polysulfides, the proportion of V^3+^ peak in the V 2p spectrum increases obviously compared with the pristine TV‐layer, disclosing that chemical redox reaction occurred between VS_4_ and polysulfides (Figure S28a,c, Supporting Information). There are also thiosulphate and polythionate species generated after interacting with polysulfides, which are usually the chemical‐adsorption intermediates of transition metal chalcogenides.^[^ ^25^ ^]^ Additionally, the peaks at low binding energies about 160.5 and 161.5 eV represent the small molecule of Li_2_S and Li_2_S_2_ (Figure S28d,b, Supporting Information), further proving the efficient catalysis effect of the TV‐layer toward polysulfides conversion.^[^ ^26^ ^]^ Therefore, when the functional TV‐PE separator was applied in LSBs, it well solves the Li dendrites formation and the polysulfides shutting problems, guaranteeing satisfactory performance. The improvement of the electrochemical performances of the LSBs with the TV‐PE and the XPS results identify the function of the TV‐layer in stabilize the sulfur cathode and lithium anode and the necessity of the electrode interface modification in regulating the reaction kinetics.

The olive LiFePO_4_ (LFP) is one of the most widely used cathode materials to couple with metallic lithium for developing the next generation of LMBs.^[^ ^27^ ^]^ Herein, LFP as cathode material was also employed in the LMBs to evaluate the TV‐PE separator. As shown in Figure 6c; and Figure S29a–c (Supporting Information), the as‐constructed LMB‐LFP batteries deliver stable cycling performance of over 400 cycles. Although the specific capacity of the LMB with PE separator differs little from that with TV‐PE separator, its Coulombic efficiency is much lower. In addition, TV‐PE separator can guarantee a more satisfactory rate performance even at 5 C (Figure S29d, Supporting Information).

Li‐rich‐layered oxides, being considered as the most promising cathode materials for future high‐energy LMBs,^[^ ^28^ ^]^ was then employed to verify the wide adaptability TV‐PE separator. First, compared with the TV‐PE separator with single side coating, bilateral modified separator (mainly discussed in this work) shows lower and more stable *R*_SEI_ and *R*_ct_ during cycling (Figure S30, Supporting Information). As seen in Figure S31 (Supporting Information), LMBs‐LMNC‐622 with TV‐PE separator deliver more stable cycling performance and higher Coulombic efficiencies than those with PE separators at low current densities of 50, 100, and 200 mA g^−1^. At a high current density of 1000 mA g^−1^, the LMB with a TV‐PE separator can achieve a specific capacity of 186 mAh g^−1^, and remain 68% of the initial capacity after 300 cycles (Figure 6d). Moreover, a good rate performance of 102 mAh g^−1^ at current density of 2000 mA g^−1^ can be achieved. When the current density turned back to 1000, 500, 200, and 100 mA g^−1^, 74.4%, 58.6%, 72.3%, and 80.5% of the original capacities recover immediately (Figure S32, Supporting Information). On the contrary, the battery with PE separator shows fast capacity decay and a worse rate performance. As being confirmed before, without appropriate regulation in the pristine PE cells, even no short circuit occurred, Li dendrites have been formed which would continuously consume the electrolyte and transform into mossy lithium lump (Figures S4 and S8, Supporting Information). This will inevitably deteriorate the electrochemical performance in the lithium full battery, finally causing fast capacity decay and low coulombic efficiency with the pristine PE separator as Figure 6c,d; and Figure S31 (Supporting Information) shown. More importantly, the soft‐packaged thin battery with the TV‐PE separator can supply about 4.5 V voltages and lighten the electronic toy in different modes (Figure 6e; and Figure S33 and Video S2, Supporting Information). Figure 6e further shows that the thin package battery can give out as high as 29 mAh capacity and stably operate for 80 cycles. Undoubtedly, the desirable electrochemical performance in the LMBs with the TV‐PE separator all illustrate the fast Li ion transportation, higher coulombic efficiency, and efficient lithium anode protection during cycling, which demonstrates the great application potential in future LMBs.

## 5 Conclusions

In summary, a competent separator with ultralight TV‐layer on polyolefin membrane was prepared to ensure the stable Li ion deposition/stripping behavior. The functional separator realizes an excellent lithium deposition and stripping for more than 3000 cycles. The kinetic property, lithium growth behavior, and SEI components of the battery fully reveal the regulation role of the functionalized TV‐layer. More importantly, the designed TV‐PE separator shows a wide compatibility in the typical LMBs systems, not only achieving a high capacity of 3 mAh cm^−2^ after 150 cycles in LSBs, but also guaranteeing high Coulombic efficiencies and stable cycling performances in the full LMBs with commercial LFP and LMNC‐622 cathode. The soft‐packaged thin batteries with the designed TV‐PE separator and LMNC‐622 cathode can stably cycle for 80 cycles, which shows a great practical application potential in future LMBs.

## 6 Experimental Section

### Raw Materials Synthesis

Graphene oxide (GO) was synthesized by the modified Hummer method.^[^ ^29^ ^]^ VS_4_ was fabricated via hydrothermal process.^[^ ^17d^ ^]^ Briefly, 0.05 m sodium orthovanadate aqueous solution was mixed with 0.3 m thioacetamide solution under continuous magnetic stirring. Then, the mixture was transferred into a teflon‐lined autoclave and heated at 180 °C for 24 h in an oven. After that, the precipitate was washed with DI water and ethanol for several times and dried at 80 °C overnight. Ta was purchased from Shanghai Macklin Biochemical Co., Ltd and used after drying in the oven at 60 °C for 24 h.

### Preparation of Functional Separators

To achieve a hydrophilic surface, the PP or PE membrane were soaked in an oxidizing solution which contains 4 m H_2_SO_4_ and 0.05 m KMnO_4_ at 30 °C for 6 h. After that, the PP or PE membrane were rinsed thoroughly with DI water and dried at 45 °C in the oven. The TV layer was composed of TA, VS_4_, and rGO with feeding weight ratio of 15:15:75 for achieving a stable functional layer. First, Ta and VS_4_ with molar ratio of 1:10 were first grinded together in ethyl alcohol. After the solvent has completely evaporated, GO solution with a few microliters of hydrazine hydrate was added into the powder mixture followed by ball‐milling for more than 12 h to obtain fine slurry. Then the slurry was coated directly on the commercial a thin PE separator (12 µm) or PP separator (25 µm) and dried at 45 °C for 24 h in a vacuum oven.

### Material Characterization

SEM was carried out on a JEOL7800 field emission SEM instrument, while transmission electro microscopy (TEM) was performed on a JEOL 2010F operating at 200 keV. Powder X‐ray diffraction (PXRD) was recorded on a Rigaku D/max 2550 PC (Cu Ka) to analyze the structure of the samples. FT‐IR tests were carried out on a NICOLET In10 (Thermo Fisher Scientific, America) infrared spectrophotometer. X‐ray photoelectron spectra (XPS) were performed on a KRATOS AXIS DLD spectrometer. Thermal‐gravimetric analysis (TGA) was conduct on a TA Instruments SDT Q600 with 10 °C min^−1^ to calculate the sulfur loading in the C/S composite and the cathode.

### Electrochemistry Characterization—Transparent Li//Li Symmetric Battery for Optical Observation

The lithium deposition experiments were operated in a homemade Li//Li symmetric model battery (as depicted in Figure 2d) at the current density of 10 mA cm^−2^. The model battery was assembled by placing two Li foils on the Cu foils. The stripping electrode was protected by the separator (PE or the TV‐PE separator). Then the Li ion transportation was regulated through this separator to the depositing side. A microscope system (SDPTOP) was applied to acquire the optical images of the lithium deposition.

### Li//Li Symmetric Cells

The Li//Li symmetric cells were assembled using Li foil with thickness of 200 µm as both working and counter electrodes with PP, TV‐PP, or TV‐PE separator, respectively. 60 µL ester‐based or carbonate‐based electrolytes were added in each cell.

### Symmetric Cell Measurements

The lithium ion conductivity (*σ*) is calculated by the following formula

(1) $$\sigma \;=\frac{L}{R\times S}\;$$

Where *L* is the thickness of the applied separator;

*R* is the high‐frequency intercept obtained from the electrochemical impedance spectroscopy;

The exchange current density (*i*_0_) is measured by linearly fitting the Tafel plots at a sweep rate of 1.0 mV s^−1^ from −200 to 200 mV.

The number of lithium ion migration (*t*_+_) was calculated based on the following formula

(2) $$t_{+}=\frac{I_{S}\left(\Delta V-I_{0}R_{0}\right)}{I_{0}\left(\Delta V-I_{S}R_{S}\right)}$$

where Δ*V* is the applied polarization voltage (10 mV);

*I*_0_ and *I*_s_ are initial current and steady‐state current, respectively;

*R*_0_ and *R*_s_ are initial resistance and steady‐state resistance, respectively.

For the LSBs, the cathode slurry composed of 70 wt% KB/S (with 86 wt % sulfur), 15 wt% conductive carbon and 15 wt% polyvinylidene fluoride (PVDF) binder with NMP solution were balling milled and coated on Al foil. The sulfur content in the cathode was about 60.2% (without considering the weight of Al foil), and Li foil was used directly as an anode. The ester‐based electrolyte was 1 m LiTFSI in a mixed solution of 1,3‐dioxolane/1,2‐dimethoxyenthane (DOL/DME) (v/v, 1:1) with 0.2 m LiNO_3_ additive. E/S ratio was controlled as 6.5 µL mg_S_^−1^ for electrode.

For the LIBs, the LFP and Li‐rich‐layered oxide cathode were also assembled with Li anode. The cathode slurry consisting of 80 wt% LFP, 10 wt% acetylene black, and 10 wt% PVDF in NMP was coated onto Al foil and dried at 110 °C for 12 h. The Li‐rich‐layered oxide cathode was prepared with the same process expect for changing the active material with the Li‐rich‐layered oxide. The electrolyte was consisted of 1 m LiPF_6_ in mixed solvent of dimethyl carbnate (DMC) and ethylene carbonate (EC) (v/v, 1:1). All coin cells were assembled in an Ar‐filled glovebox (<0.5 ppm of O_2_ and H_2_O).

### Computational Method

The simulation module was composed of Li surface and the designed TV layer or PE with liquid electrolyte impregnated inside. The thickness of the functional layer or PE was fixed at 5 µm as the square with computational grids in Figure S12 (Supporting Information) using the electrodeposition module in COMSOL. The overall Li ion transport and mobility parameters of the Li symmetrical batteries with TV‐PE or PE separator were set according to the results in Tables S1 and S2 (Supporting Information).

### Mechanical Performance Examinations

The 180° peeling test of the TV‐PE separator was conducted according to Figure S22a (Supporting Information). The commercial polyimide tape was stuck on the TV layer with width of 10 mm. A universal tension machine was used to stretch the tape and the TV‐PE terminals at the pulling speed of 1 mm min^−1^ and the force–distance curve was recorded at the same time. The nanoindentation tests were conducted on the Nano Indenter II MTS with the applied load of 10 mN.

## Conflict of Interest

The authors declare no conflict of interest.

## Author Contributions

The manuscript was written through contributions of all authors. All authors have given approval to the final version of the manuscript. Q.Z., R.W., and C.X. conceived the idea and wrote the manuscript. R.W. and C.X. provided financial support through grant application. Q.Z. and R.W. performed the TEM, XPS, FT‐IR, and data analysis. Q.Z. and X.H. helped the material characterization of SEM and XRD tests. Q.Z. and Y.W. conducted the theoretical simulations. Q.Z., G.L., Z.Y., Q.L., and F.P. helped the electrochemical test and data analysis. Q.L. and X.Y. provided the LMNC‐622 cathode material. All authors participated in discussing and approved the final manuscript.

## Supporting information

Supporting Information

Supplemental Video 1

Supplemental Video 2

## Data Availability

The data that support the findings of this study are available on request from the corresponding author. The data are not publicly available due to privacy or ethical restrictions.
