# Supplementary material for: Functionalized 12 µm Polyethylene Separator to Realize Dendrite‐Free Lithium Deposition toward Highly Stable Lithium‐Metal Batteries
Source: Adv Sci (Weinh). 2022 Mar 7;9(13):2102215. doi: 10.1002/advs.202102215 (PMC9069191; doi:10.1002/advs.202102215)
Supplement: Supplementary file 1 — Supporting Information [file ADVS-9-2102215-s001.pdf]

ADVANCED SCIENCE
Open Access

Supporting Information

for *Adv. Sci.*, DOI 10.1002/advs.202102215

Functionalized 12 μm Polyethylene Separator to Realize Dendrite-Free Lithium Deposition toward Highly Stable Lithium-Metal Batteries

Qiannan Zhao, Ronghua Wang, Xiaolin Hu, Yumei Wang, Guanjie Lu, Zuguang Yang, Qiwen Liu, Xiukang Yang, Fusheng Pan and Chaohe Xu**

Supplementary Information for

Functionalized 12 μm Polyethylene Separator to Realize Dendrite-Free Lithium Deposition towards Highly Stable Lithium-Metal Batteries

Qiannan Zhao[1], Ronghua Wang[2,*], Xiaolin Hu[1], Yumei Wang[1,3], Guanjie Lu[1], Zuguang Yang[1], Qiwen Liu[4], Xiukang Yang[4] Fusheng Pan[2,5], and Chaohe Xu[1, 5,*]

[1] *College of Aerospace Engineering, Chongqing University, Chongqing, 400044, P.R. China*

[2] *College of Materials Science and Engineering, Chongqing University, Chongqing, 400044, P.R. China*

[3] *National University of Singapore (Chongqing) Research Institute, Chongqing, 401123, P.R. China*

[4] *Hunan Province Key Laboratory of Electrochemical Energy Storage and Conversion, School of Chemistry, Xiangtan University, Xiangtan 411105, P.R. China*

[5] *National Engineering Research Center for Magnesium Alloys, Chongqing University, Chongqing, 400044, P.R. China*

KEYWORDS: Lithium metal batteries, functionalization, composite separator, lithium anode, dendrite-free deposition

***Corresponding Author**

Email: xche@cqu.edu.cn (C. Xu); wangrh@cqu.edu.cn (R. Wang)

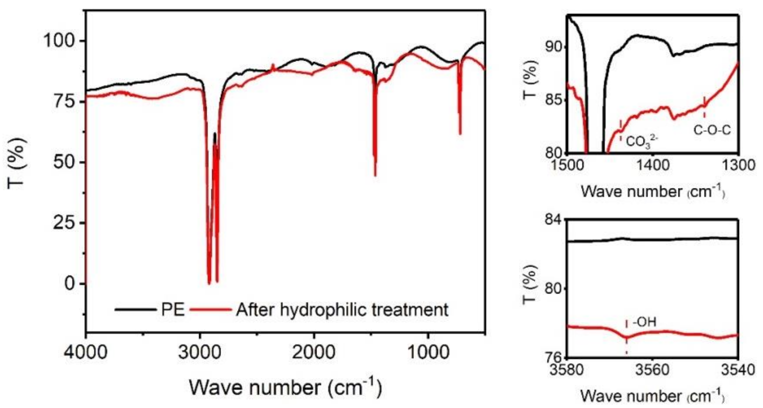

Figure S1. FT-IR spectrums of PE separator before and after hydrophilic treatment.

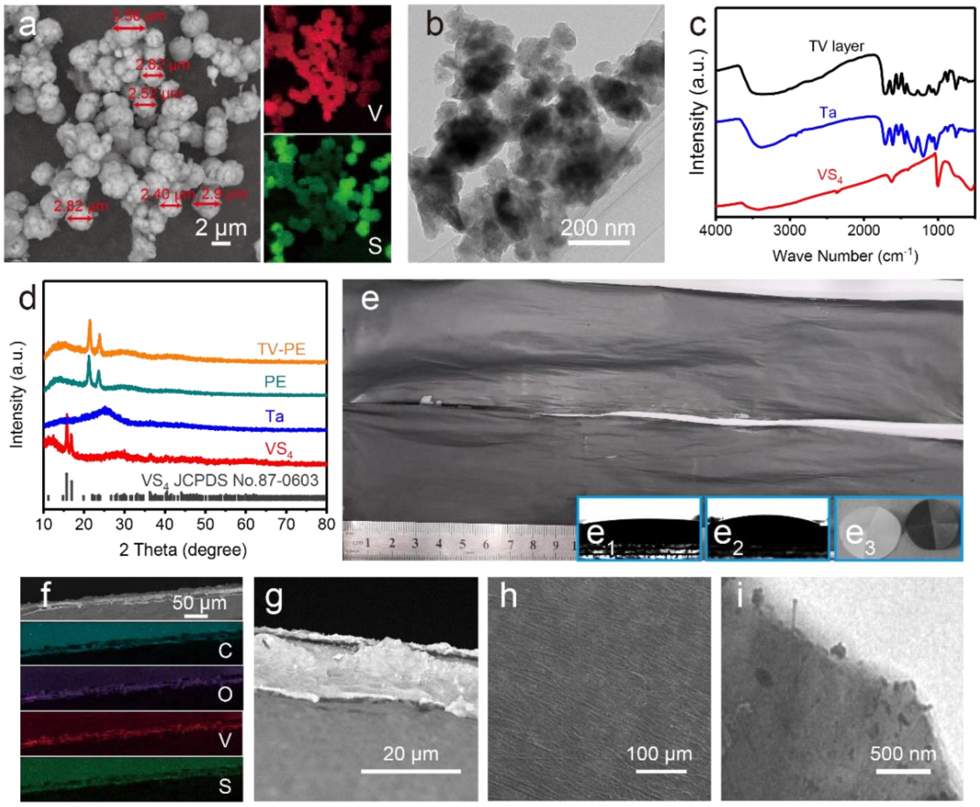

Figure S2. Micro-morphologies of VS_4 (a) SEM image and corresponding EDS mapping, (b) TEM image after grinding and ultrasonic treatment. (c) FT-IR spectrums of VS_4, Ta and the TV layer scraped from the TV-PE composite separator. (d) XRD results of VS_4, Ta, PE membrane and TV-PE composite separator. (e) Photograph of two pieces of the as-prepared TV-PE separator. The insets of e_1 and e_2 show the wetting ability of TV-PE separator for the ester and carbonate electrolyte, while e_3 shows the structural stability of the functional TV layer on the PE matrix after folding. Micro-structures of the TV-PE composite separator, (f) cross-section SEM image and EDS mapping with low magnification, (g) cross-section SEM

image with high magnification to measure the thickness of the functional TV layer. (h) Top-view SEM image and the TEM image the TV-layer.

The as-prepared VS_4 showed a sphere-like morphology with a diameter of about 2 μm with rough surface, which is closely assembled by tiny nanoparticles of 100~200 nm (**Figure S2**a-**S2**b). FT-IR spectra of the TV-layer display similar characteristic peaks as the Ta, while no obvious peaks of VS_4 are observed (**Figure S2**c). XRD patterns confirm that VS_4 is the monoclinic phase (JCPDS 87-0603) while Ta shows an amorphous state with only one plain peak as seen in **Figure S2**d. The reason that VS_4 and Ta are not detected in the TV-layer for the FT-IR spectra and XRD tests could attribute to their small proportion. The insets of **Figure S2**e_1 and **S2**e_2 reveal the good electrolyte (ester- and carbonate-based electrolyte) wettability of the TV-PE separator. After being folded several times, almost no powder exfoliated, further illustrating the good adhesion between the TV-layer and the PE basement membrane (inset **Figure S2**e_3). The TV-PE separator can maintain the structure stabilization well, and is feasible for large scale production as shown in **Figure S2**e. The micro-morphology of the as-prepared TV-PE separator shows that most of VS_4 particles with decreasing size after vigorous grinding are well wrapped by the winkled rGO nanosheets in the TV-layer (**Figure S2**f-**S2**h). From this view, the well wrapped VS_4 and Ta also may lead to their almost invisible signals in the FT-IR and XRD results, respectively.

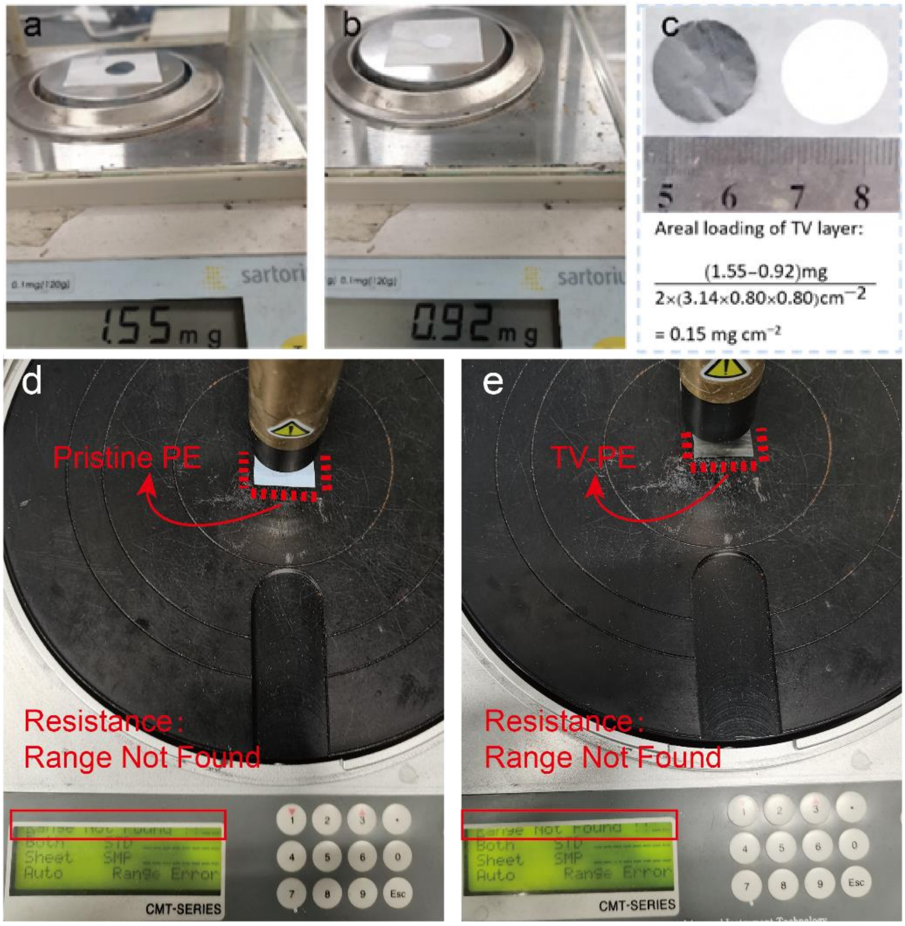

Figure S3. The mass of (a) TV-PE membrane, (b) PE membrane and the corresponding calculation of the TV areal loading in (c). Electrical conductivity tests of (d) pristine PE and (e) TV-PE separator.

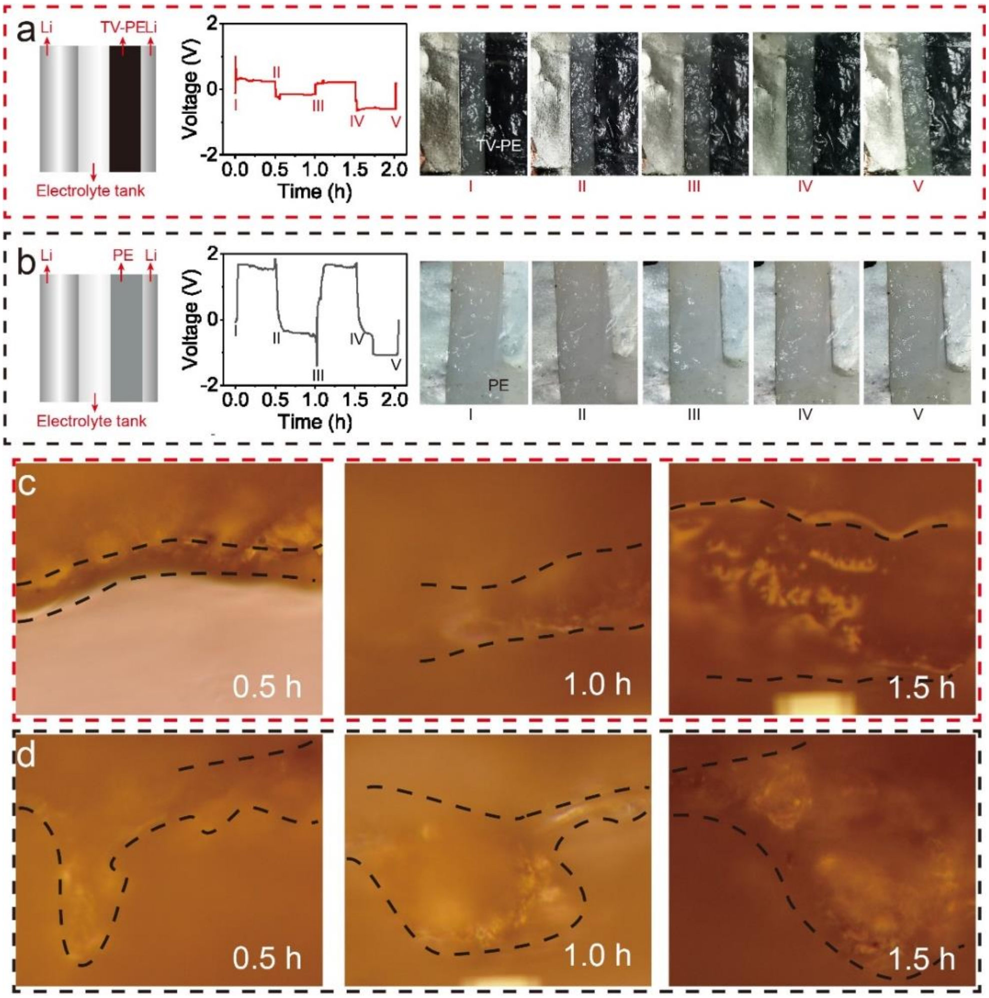

Figure S4. In-situ optical observation of the lithium deposition process with different separators. Lithium deposition and stripping behavior and recorded electronic photos at different I-V stages in the voltage profiles with (a) TV-PE separator and (b) PE separator. Optical images of the Li anode for 1.5 h deposition regulated by (c) TV-PE and (d) PE separator which were recorded by a microscope system. The current density was set as 10 mA cm^{-2} in the optical lithium deposition process.

Firstly, the application of the TV-PE separator drastically reduced the overpotential of the lithium deposition to 0.33 V vs. 1.67 V of the pristine PE separator under high current density of 10 mA cm^{-2}. During cycling, the lithium deposition/stripping terminal regulated by a TV-PE separator kept smooth and lustrous in contrast with the gradually dark and mossy lithium terminals regulated by pristine PE separator.

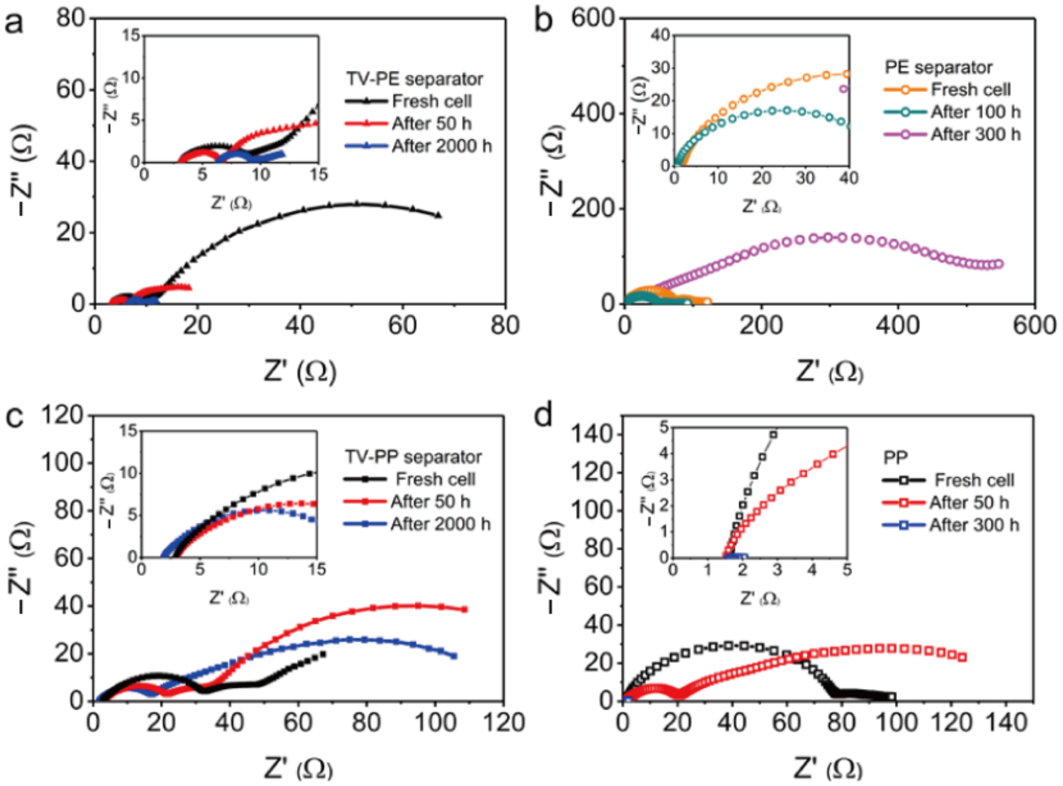

Figure S5. EIS results of Li-Li symmetric cells before and after cycling with (a) TV-PP, (b) PP, (c) TV-PE and (d) PE separator.

During the long cycling duration, the Li//TV-PP//Li symmetric battery showed a much more stable impedance property than that of the Li//PP//Li symmetric battery (**Figure S5**c-**S5**d).

Table S1. Impedance parameters of the Li symmetrical batteries with TV-PE and PE separators.

Separator	σ (S cm^{-1})	D_{Li^+} (cm^2 s^{-1})
TV-PE	1.8×10^{-4}	1.16×10^{-12}
PE	1.5×10^{-5}	3.46×10^{-12}

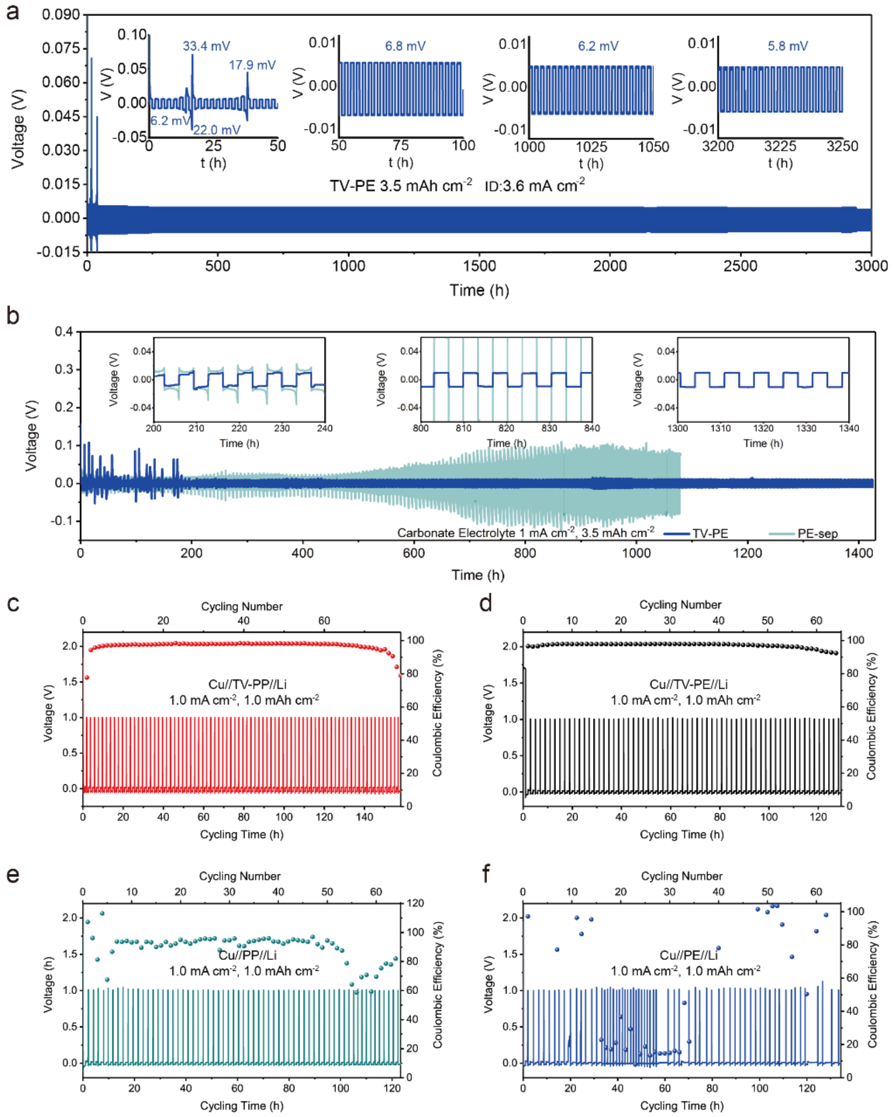

Figure S6. (a) Electrochemical performances of Li-Li symmetric cells with PE and TV-PE separators using ester electrolyte with capacity is 3.5 mAh cm^{-2} at the current density of 3.6 mA cm^{-2} and (b) carbonate electrolyte composed of 1 M $LiPF_6$ in mixed solvent of DMC and EC (v/v, 1:1) with capacity is 3.5 mAh cm^{-2} at the current density of 1 mA cm^{-2}. (c) Electrochemical performance of the Cu-Li half cells with (c) designed TV-PP, (d) designed TV-PE, (e) pristine PP, and (f) pristine PE separator using ester electrolyte with capacity is 1.0 mAh cm^{-2} at the current density of 1.0 mA cm^{-2}.

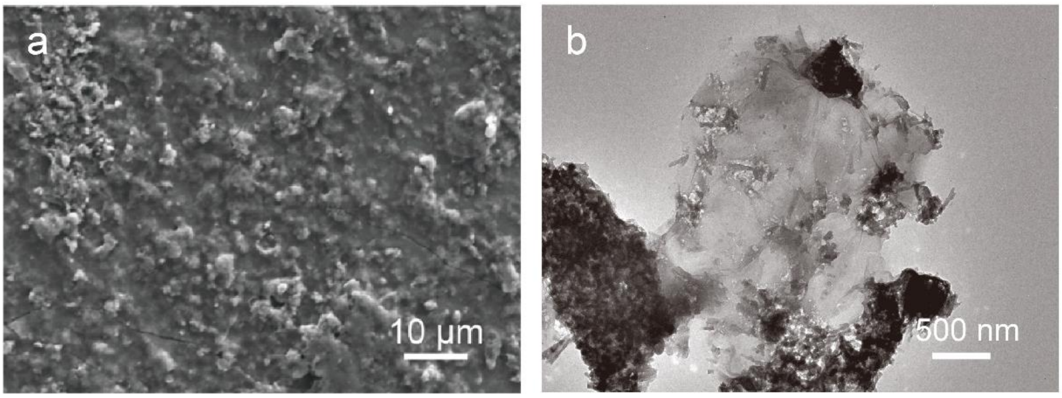

Figure S7. SEM image (a) and TEM image of TV-functional layer in the Li//TV-PE//Li symmetric battery after cycling 400 h at current density of 2.6 mA cm^{-2} with 3.5 mA cm^{-2} deposition capacity.

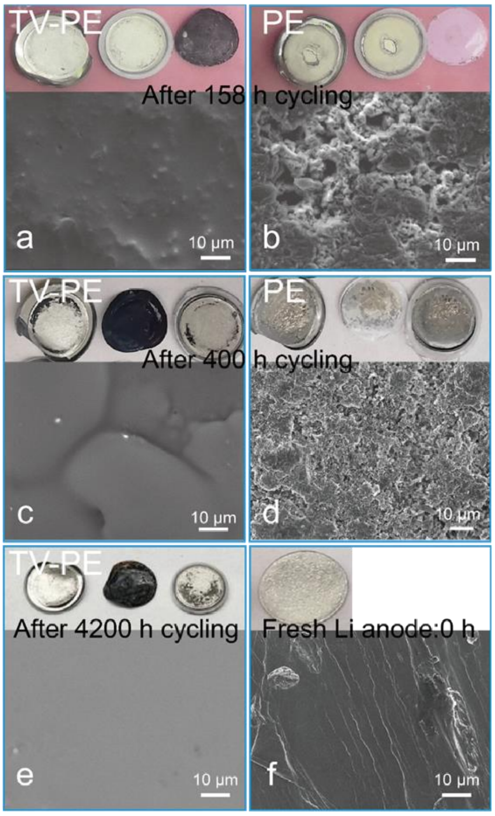

Figure S8. Li anodes changes before and after cycling. The disassembled Li anode (a-b) after 158 h cycling, (c-d) after 400 h cycling at current density of 2.6 mA cm^{-2} with 3.5 mA cm^{-2} deposition capacity, (e) after 4200 h cycling at current density of 3.6 mA cm^{-2} with 3.5 mA cm^{-2} deposition capacity, (f) Original Li anode. The batteries of (a), (c), (e) were with TV-PE separators and (b), (d) were with PE separators.

Compared with the macro-view of the fresh Li anode in **Figure S8**f, the cycled Li anode regulated by TV-PE separator keeps shiny slivery state all the time, even after 4200 h cycling (**Figure S8**a, **S8**c, and **S8**e). The cycled Li anodes show dense and smooth micro-morphologies as the cycling life goes on from 158, 400 to 4200 h. The uniform and smooth micro-morphologies of the cycled Li anode also illustrated the Li ion regulation function of the TV-PE separator as some micro-protuberances and defects obviously exist on the fresh Li surface (**Figure S8**f). However, the Li anodes with the PE separator after cycling show passivated and pulverizing macro and micro-surface.

This ex-situ detection process agrees well with the simulation results that TV-PE separator can alleviate the effect of defects and regulate the Li ion distribution during Li deposition and stripping process.

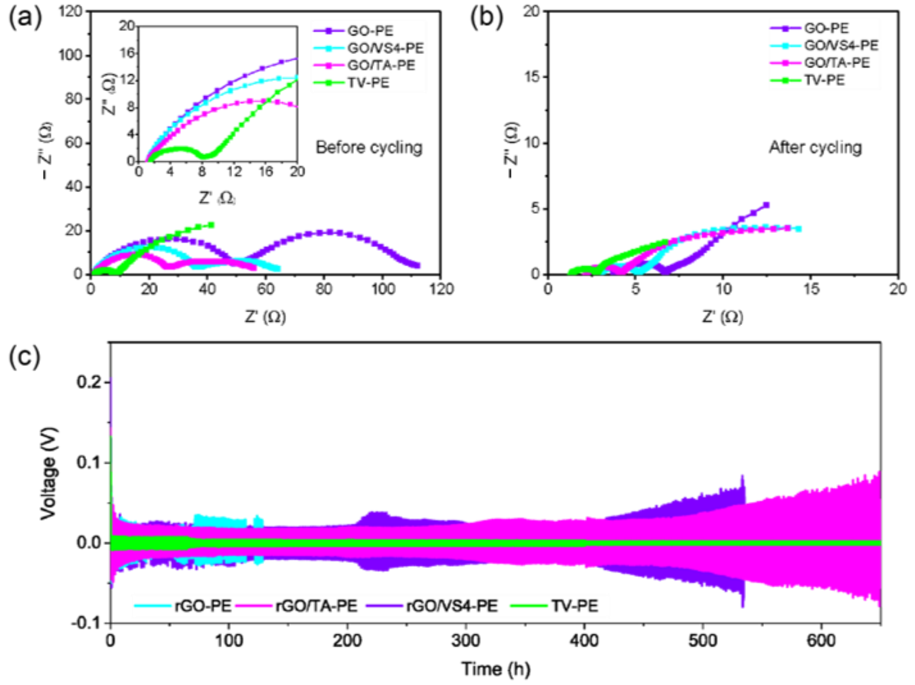

Figure S9. EIS results of the fresh (a) and cycled (b) lithium symmetric batteries in (c). (c) The cycling performance with rGO-PE, rGO/VS_4-PE, rGO/TA-PE, and TV-PE separator. The batteries were cycled at current density of 2.6 mA cm^{-2} with 3.5 mA cm^{-2} deposition capacity.

As **Figure S9**a-**S9**b shows, the battery resistance before and after cycling follows the

trends: TV-PE<rGO/TA-PE<rGO/VS_4-PE<rGO-PE, which is the same as the trend of overpotential and the cycling stability of lithium deposition and stripping (**Figure S9**c). These results identify the function of TA which could enhance the ionic conductivity of the battery and the synergistic function of TA, VS_4, and rGO wrapper in regulating the interfacial electrochemistry of the battery.

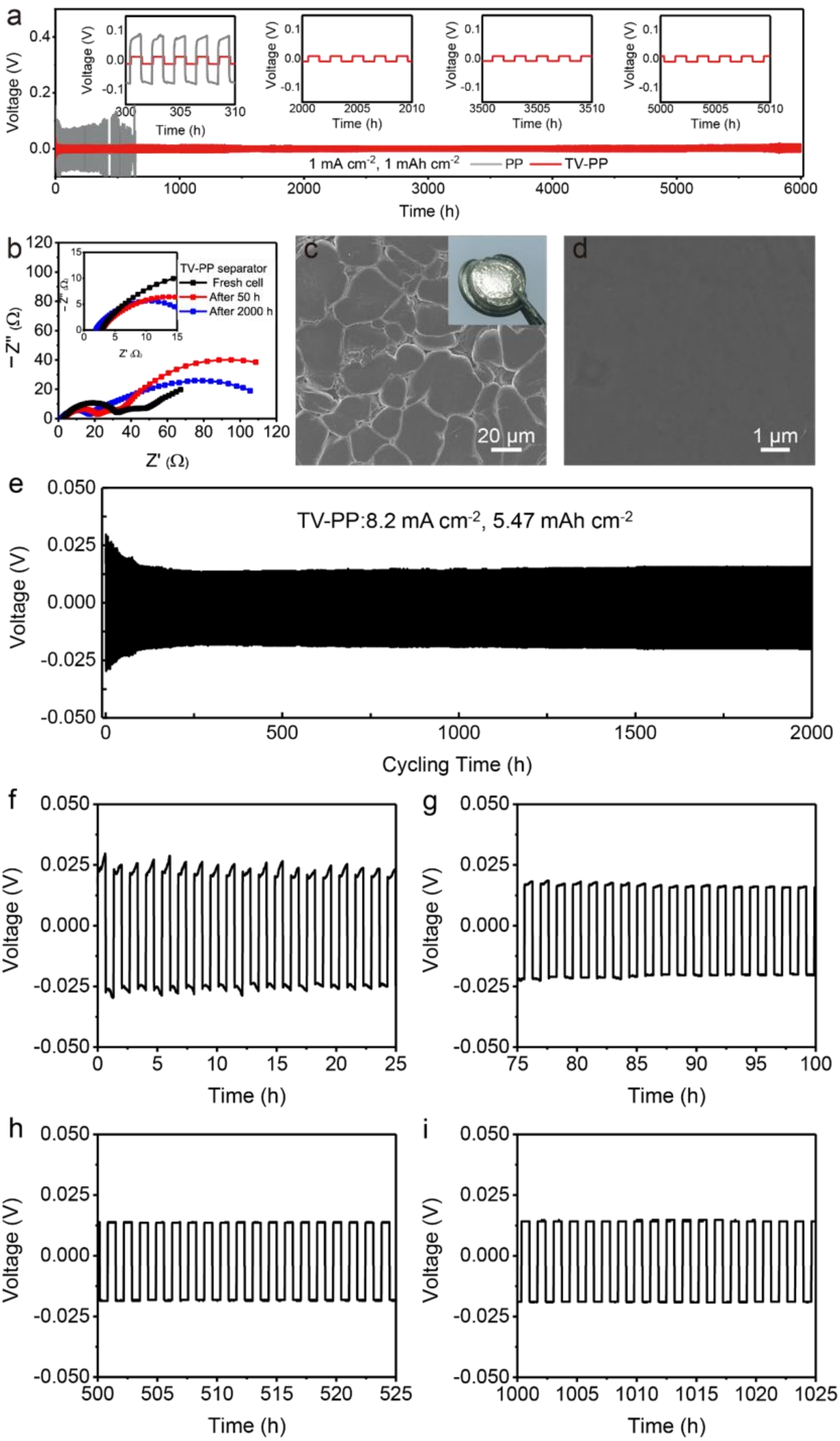
a
Voltage (V)
0.4
0.2
0.0
1 mA cm-2, 1 mAh cm-2
PP
TV-PP
Time (h)
0
1000
2000
3000
4000
5000
6000
Voltage (V)
0.1
0.0
-0.1
300
305
310
2000
2005
2010
3500
3505
3510
5000
5005
5010
b
-Z'' (Ω)
Z' (Ω)
TV-PP separator
Fresh cell
After 50 h
After 2000 h
0
20
40
60
80
100
120
c
20 μm
d
1 μm
e
TV-PP:8.2 mA cm-2, 5.47 mAh cm-2
Voltage (V)
0.050
0.025
0.000
-0.025
-0.050
0
500
1000
1500
2000
Cycling Time (h)
f
g
h
i
Voltage (V)
Time (h)
0
5
10
15
20
25
75
80
85
90
95
100
500
505
510
515
520
525
1000
1005
1010
1015
1020
1025

Figure S10. Electrochemical performance of Li-Li symmetric cells with PP or TV-PP membrane as separator. (a) Ultra-long cycling profiles at a current density of 1 mA cm^{-2} for 1 mAh cm^{-2}. (b) EIS results of the Li-Li symmetric cell with TV-PP separator after different cycles. (c) Low magnification and (d) high magnification SEM images of Li anode in the Li//TV-PP//Li symmetric cells after 6000 h cycling. Inset in (c): digital photo of the dis-assembled Li anode. (e-i) Long cycling performance Li-Li symmetric cells with TV-PP separator at high current density of 8.2 mA cm^{-2} and deposition capacity of 5.47 mAh cm^{-2}. (f-i) are partial enlarged views of (e).

The Li-Li symmetric battery with original commercial PP separator can only cycle for about 340 h at low current density of 1 mA cm^{-2} with significant fluctuation during cycles and an abruptly short-circuit (**Figure S**10a), indicating the unstable electrochemical interface and dendritic punctuation.

Noted the voltage profiles of symmetric lithium batteries are relatively flat rather than classic "arch" ones in this work, which indicates barely lithium whisker nucleation or dendrites accumulation during cycling. This mild lithium depositing and stripping phenomenon of flat voltage profiles and small over potential usually appears in good mechanical electrode interface system with fast electronic and ionic behavior and has been widely discussed in recent literatures [1].

Table S2. The exchange current density (i_0) and lithium ion migration number (t^+) of the Li symmetrical batteries with different separators.

Separator	i_0 (mA cm^{-2})	t^+
PE	0.50	0.48
TV-PE	0.29	0.68
PP	0.26	0.30
TV-PP	0.14	0.59

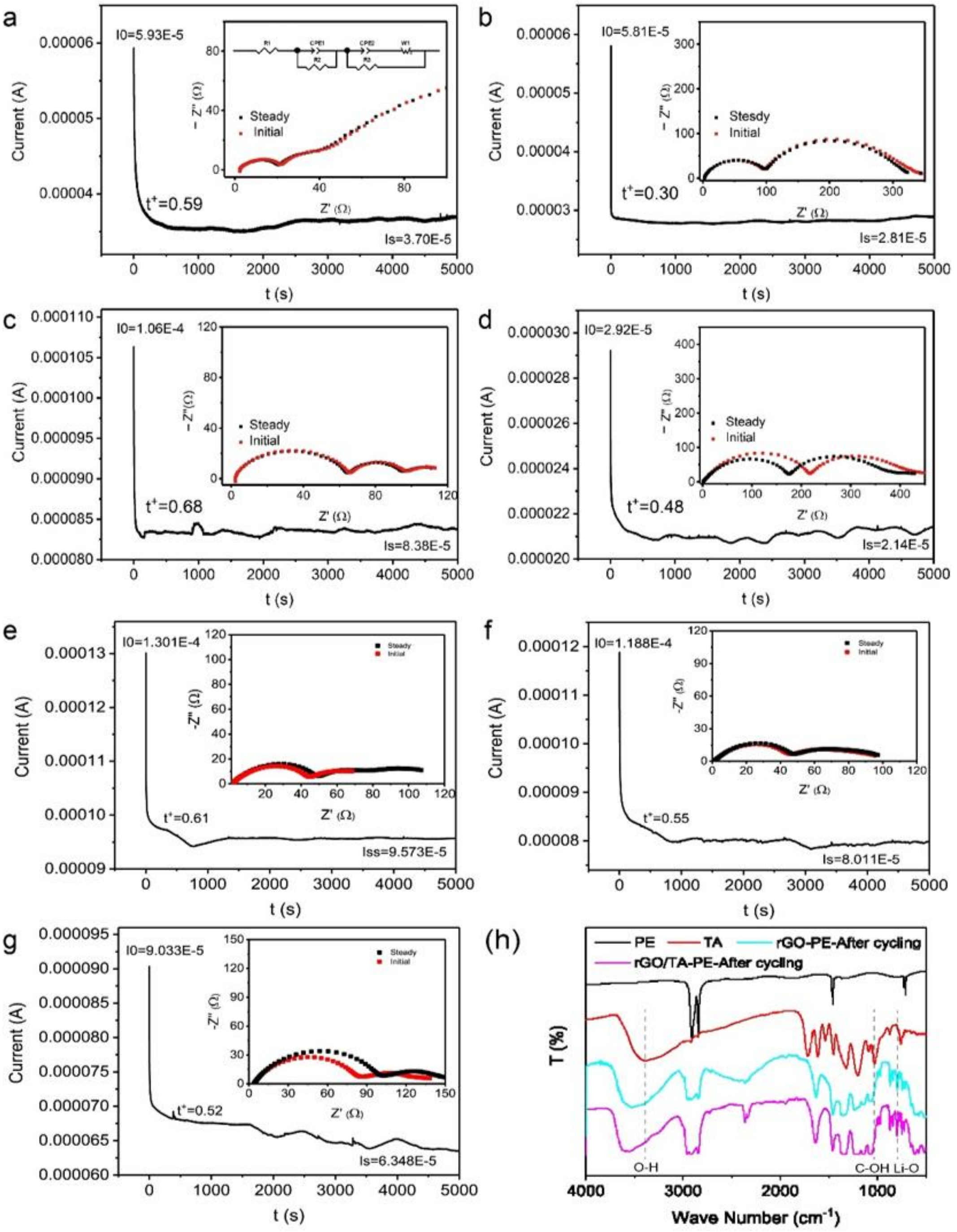

Figure S11. Lithium ion migration number (t^+) of in the lithium symmetric batteries with (a) TV-PP, (b) PP, (c) TV-PE, (d) PE separator, (e) rGO/TA-PE, (f) rGO/VS_4-PE, and (g) rGO-PE separator. (h) FT-IR spectra of pristine PE, TA powder, rGO-PE after cycling, and rGO/TA-PE after cycling.

The Lithium ion migration number (t^+) is an important parameter reflecting the Li ion transport property. It is closely related to the Li deposition behavior and the high charging rate/energy density performances of batteries. For the liquid electrolyte with the polyolefin

separator combination, the value of t^+ is about 0.2-0.5. As shown in **Figure S10a-S10d**, the t^+ of PP separator-25 μm and PE separator-12 μm was remarkably increased from 0.30 and 0.48 to 0.59 and 0.68 after TV layer modification, respectively. Thus, the functionalized TV-PP and TV-PE separators can supply efficient cation transfer capacity and regulate the uniform Li deposition. In addition, when they are applied in the full LMBs, they could further guarantee the high rate performance and high energy density delivering.

From the Sand's formula, we could know that the lithium dendrite will nucleate when the cation concentration at the electrode surface decreases to zero and the current density becomes over-limiting. Thus, improving t_{Li}^+ is a feasible way to lower the current density and eventually avoid lithium dendrites formation [2]. As the TV-layer modification could greatly improve t_{Li}^+ and decrease the interfacial resistance which is key points in regulating the lithium deposition, it makes the dendrite-free lithium deposition come true.

Sand's formula:

$$t_{sand} = \Pi \mathrm{Dapp} \frac{(z_c c_0 F)^2}{4(J t_a)^2}$$

Where, Dapp is the apparent diffusion coefficient, Zc is the cationic charge number, c_0 us the bulk salt concentration, F is the Faraday's constant, J is the current density.

FT-IR spectra of the pristine PE separator, TA, cycled rGO-PE, and cycled rGO/TA-PE after cycling in **Figure S11** explore the interaction between TA and lithium. After cycling, the blue shift of C-OH to 1215 cm^{-1} in cycled rGO-PE and cycled rGO/TA-PE vs. 1205 cm^{-1} in TA. Additionally, Li-O at 580 cm^{-1} appears in the cycled rGO/TA sample which could be attributed to the Li-alkoxide [3]. Compared with TA, -OH signals at around 3500 cm^{-1} still exist in the cycled rGO-PE and cycled rGO/TA-PE separator samples with obvious blue shifts (**Fig. S11**h). Combined with the XPS tests of Li-O specie existence on the Li surface in **Fig. 5**d, we could conclude that TA on the TV layer surface can interact with the lithium and generate Li-alkoxide to modify the SEI on the lithium anode.

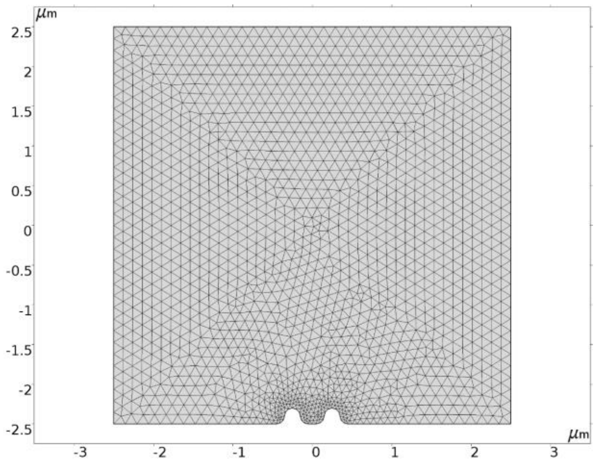

Figure S12. Theoretical simulation model with mesh.

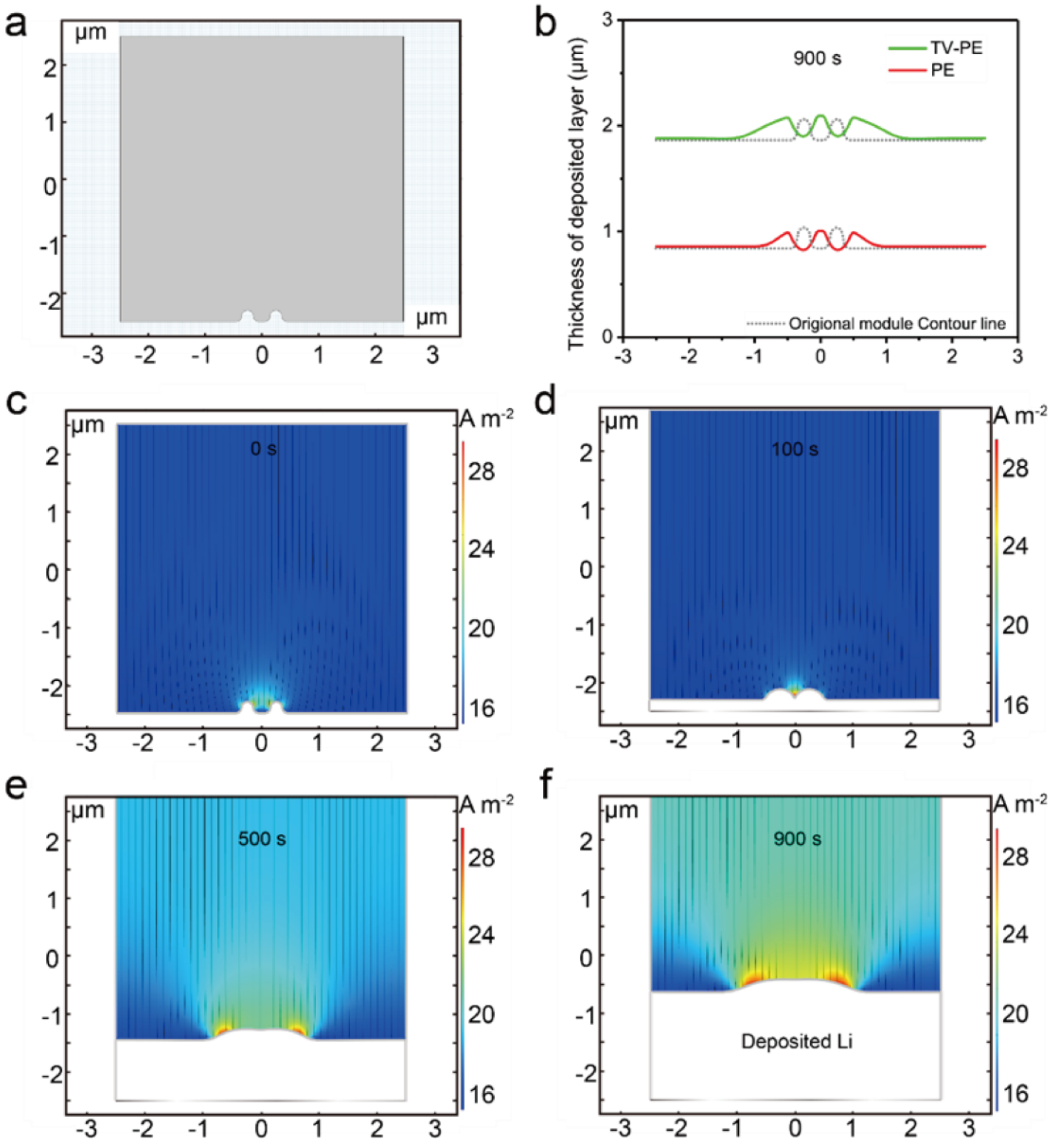

Figure S13. Theoretical simulation of lithium deposition on the Li anode surface with bulge

defects with TV-PE and PE separators. (a) Simulation model. (b) Relative Li deposition thickness after 900 s with TV-PE and PE separator, the light grey profiles represent the original surface profile of the Li surface. (c-f) The lithium ions flux distribution and deposition states at 0, 100, 500 and 900 s, respectively.

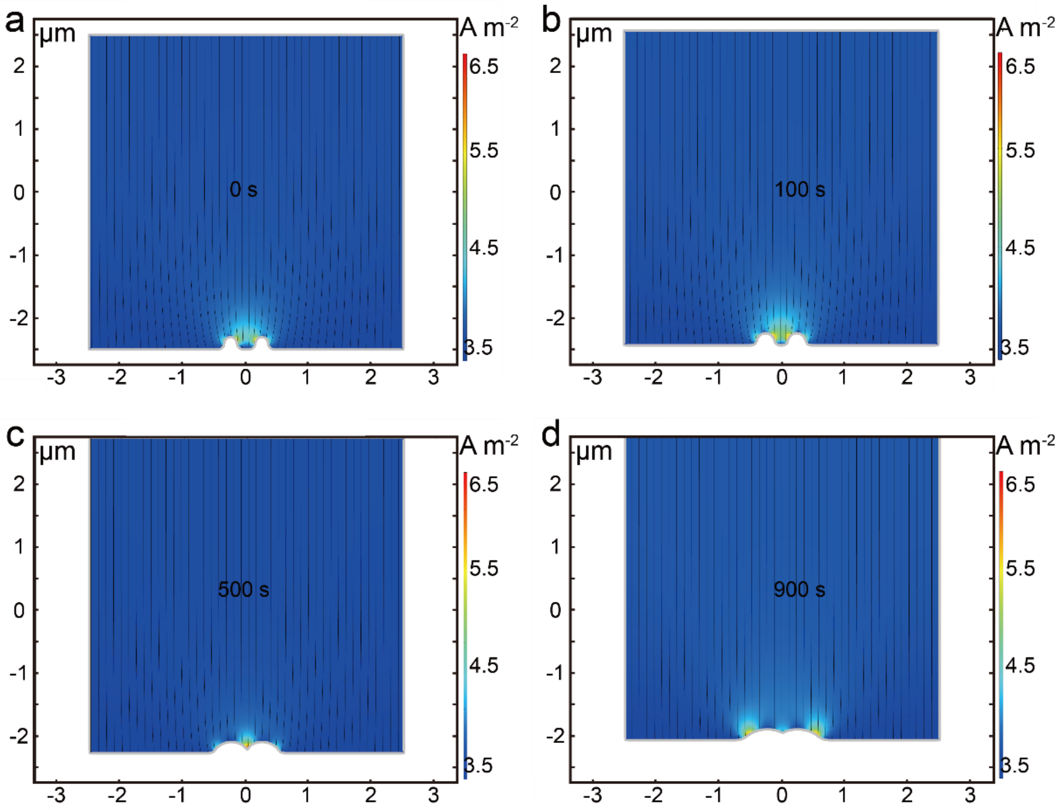

Figure S14. Theoretical simulation results of the Li deposition behavior with PE separator at different times.

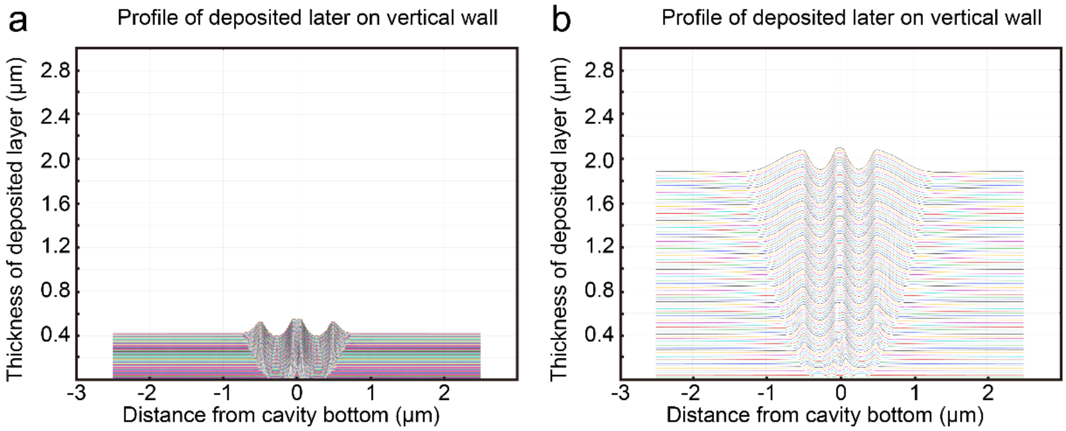

Figure S15. Contour profiles of thickness of deposited Li layer on the vertical wall with (a) PE and (b) TV-PE separator.

The bulges with 200 nm height in the simulation model was designed according to actual defects (**Figure S8**f) to represent the roughness disturbance on both fresh and cycled Li surface. The fast Li ion transport properties of the TV-PE separator regulate the uniform Li ion distribution and deposition, especially at the corner region of the bulges, attenuating the sharp nuclei and the dendrites formation potential (**Figure S13**b and **Figure S15**).

As a result, the angular tips were adjusted into a plain profile by the designed TV-PE separator during the deposition process (**Figure S13c- S13f**). However, the obvious tip defects still exist in the PE module (**Figure S14**). These simulations reveal the function of the separators in regulating the Li ion deposition behavior, even with some local defects.

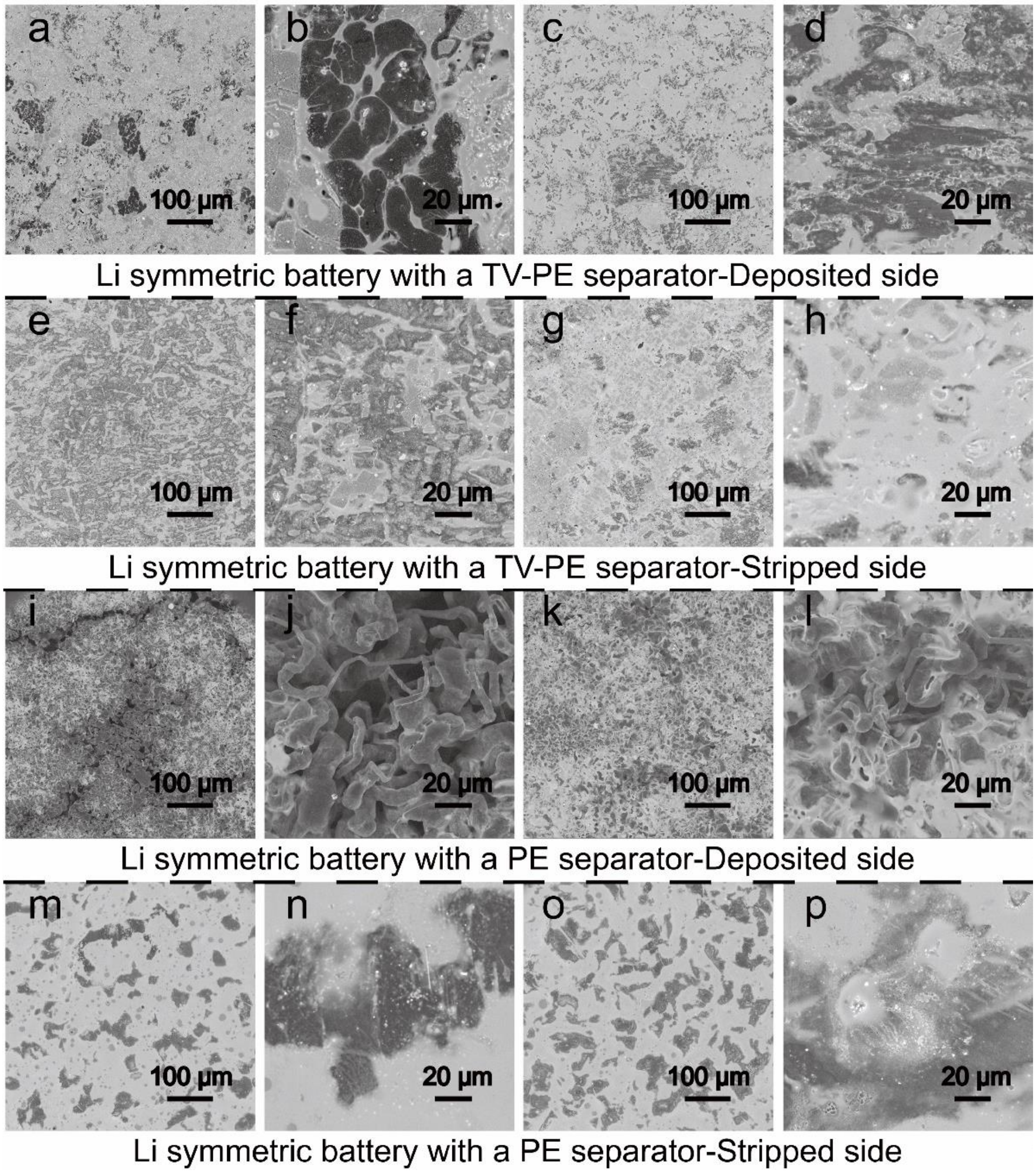

Figure S16. SEM images of the electrodes in Li symmetric batteries after continuous deposition with a potential fluctuation in **Figure 4**e **and 4**f. (a-d) lithium deposited state, (e-h) lithium stripped state regulated by the TV-PE separator. (i-l) lithium deposited state, (m-p) lithium stripped state with the PE separator.

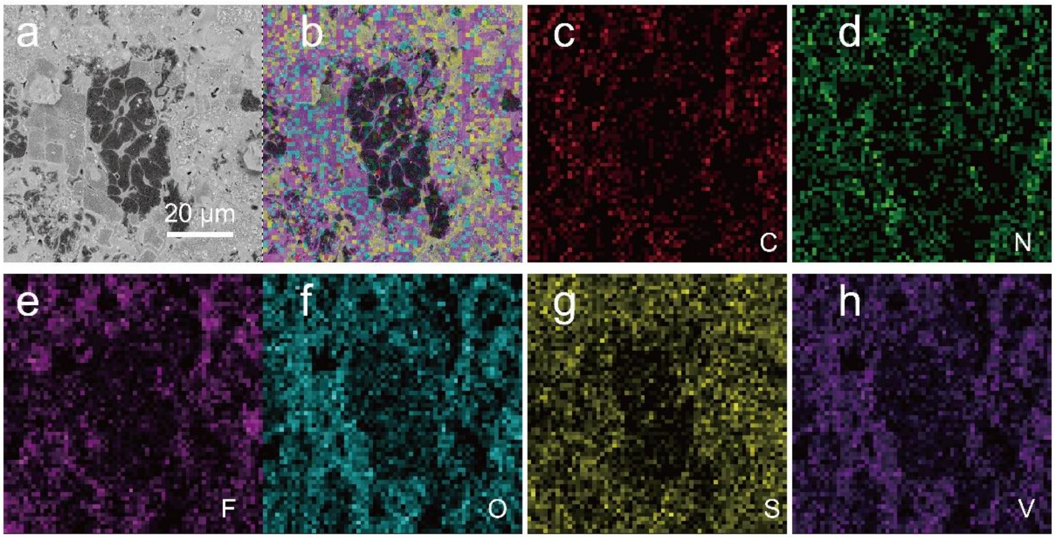

Figure S17. Element mappings of the deposited Li electrode protected with a TV-PE separator.

Representative morphologies of the cycled Li anode were chosen from the overall morphology in **Figure S16-S17**. The TV-PE separator regulates the lithium to grow horizontally into dense cobblestone-like lithium (**Figure S16**a-**S16**b) as uncertain disturbance appears after high-capacity cycling, in addition to the smooth Li deposition surface (**Figure S16**c-**S16**d). EDS mappings further confirm the existance of the cobblestone-like lithium. On the contrary, under such high capacity and long-time cycling, the deposited Li terminal coupled with a PE separator was fully covered with mossy Li dendrites. It is inevitably to form some large cracks with such loose Li deposition (**Figure S16**i) and induce the final pulverization of the Li electrode. The loose dendrites were uneven with diameter from several hundreds of nanometers to tens of micrometers (**Figure S16**c-**S16**l).

On the stripped side, the Li surface with the TV-PE is much flatter and smoother than that with the PE separator (**Figure S16**e-**S16**h and **S16**m-**S16**p), reflecting that Li ion transport of TV-PE separator is more uniform than that of original PE separator.

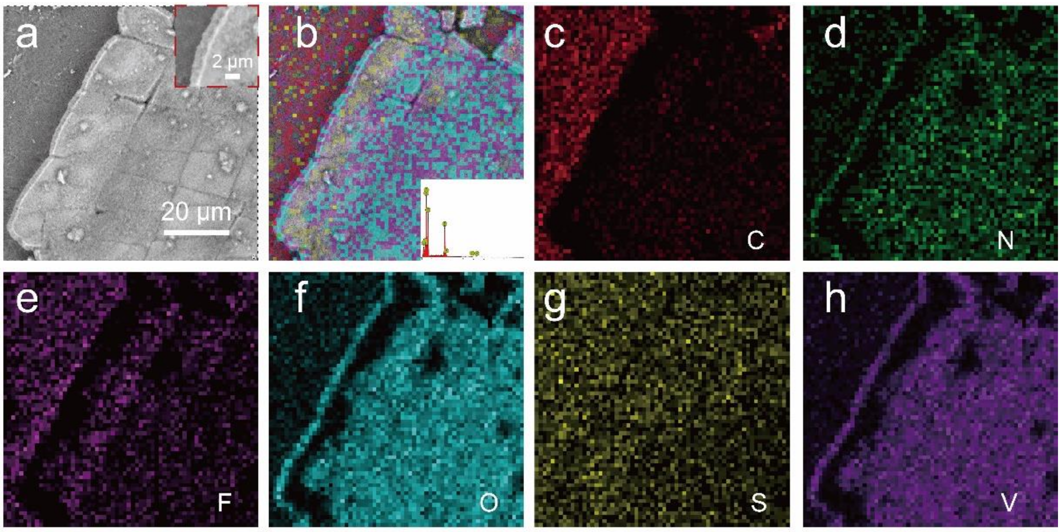

Figure S18. Element mappings of the TV-PE separator after cycling.

The thickness of TV layer after cycling (**Figure S18**) is basically the same as that before cycling (**Figure S2**g), but some cracks appear due to the large lithium deposition stress.

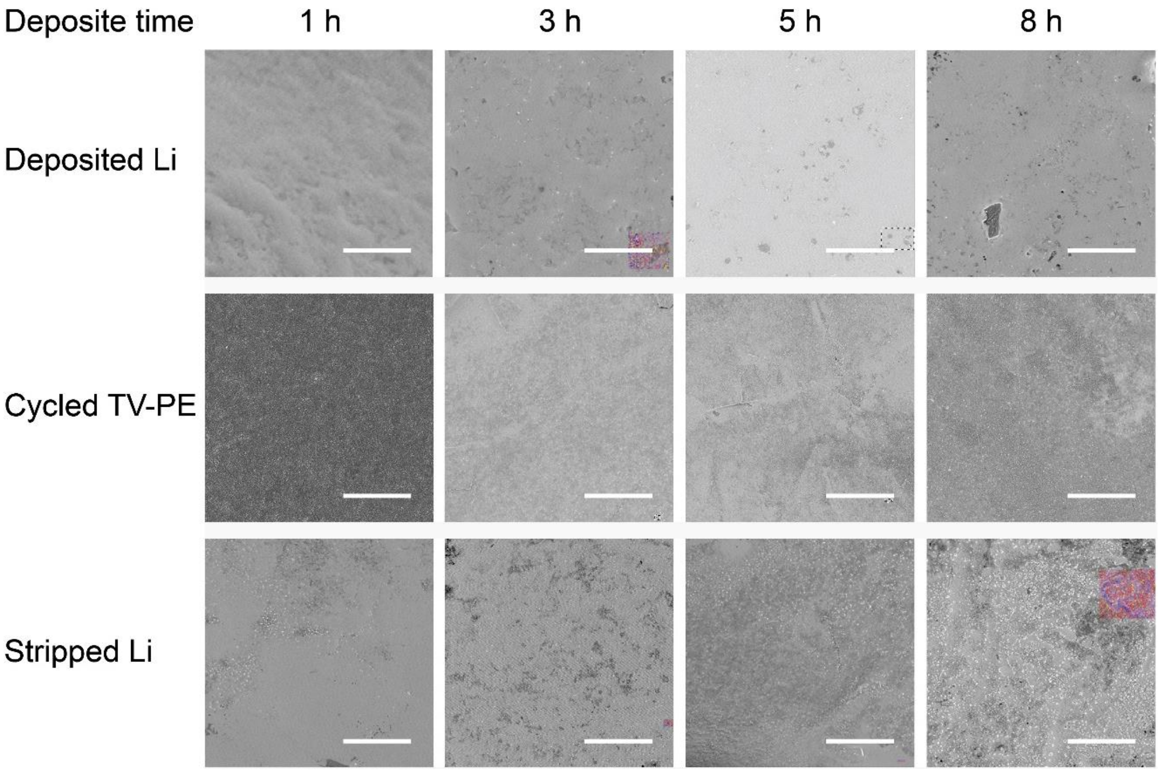

Figure S19. SEM images of lithium deposition/stripping morphology and the corresponding cycled TV-PE separator in Li symmetric batteries after 1, 3, 5 and 8 h. The current density is 1.5 mA cm^{-2}. The scale bar in the figure is 80 μm.

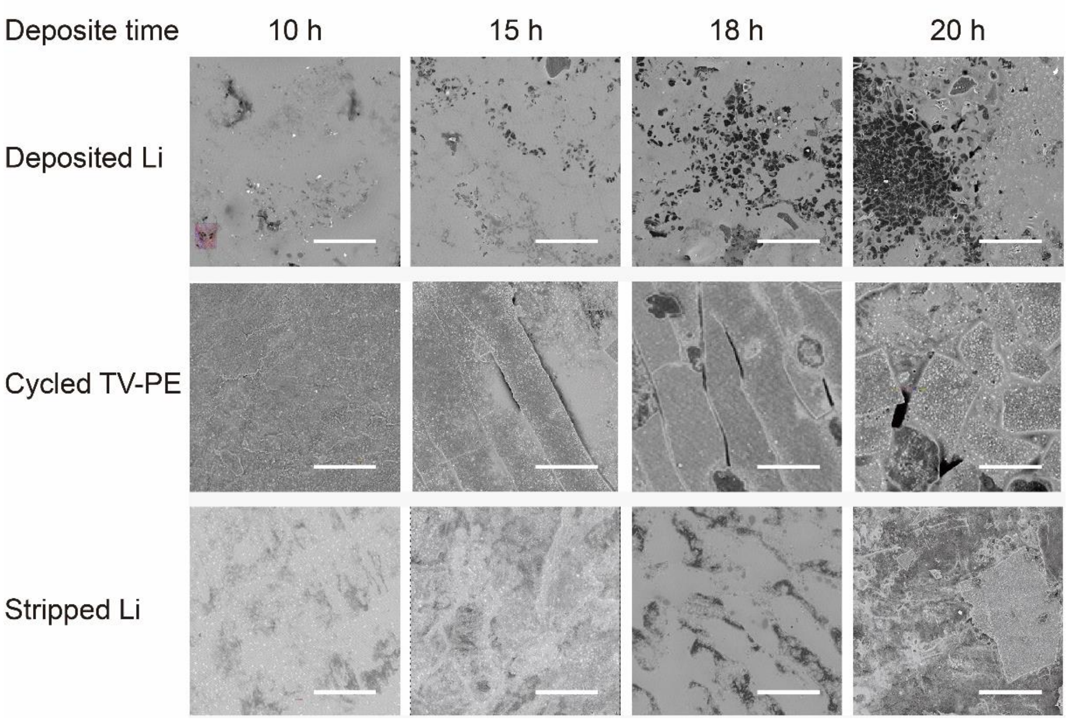

Figure S20. SEM images of lithium deposition/stripping morphology and the corresponding cycled TV-PE separator in Li symmetric batteries after 10, 15, 18 and 20 h. The current density is 1.5 mA cm^{-2}. The scale bar in the figure is 80 μm.

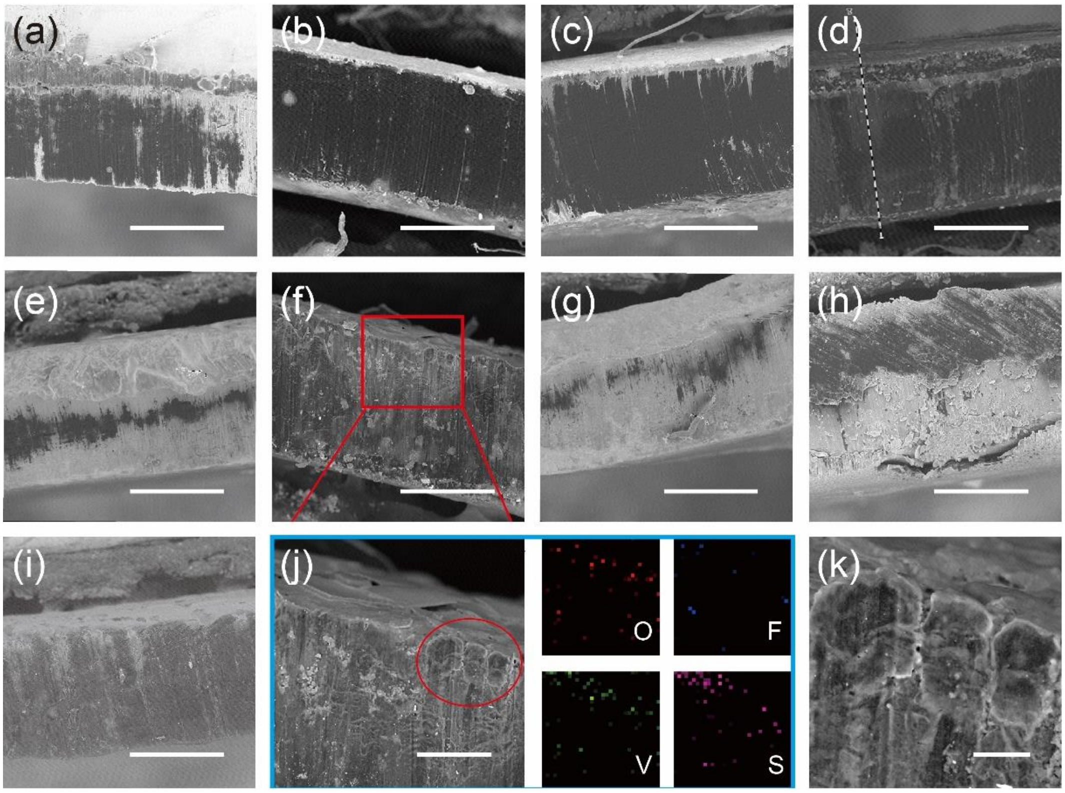

Figure S21. SEM images of deposited lithium with the TV-PE separator in Li symmetric

batteries after (a) 1, (b) 3, (c) 5, (d) 8, (e)10, (f) 15, (g) 18, (h) 20 h and (i) the fresh lithium foil. (j) The high magnification image in (f) showing some TV layer adhere to the deposited lithium surface. (k) SEM image of the red circle region in (j) clearly showing the cross-section image of the cobblestone lithium deposition. The scale bar is 200 μm in (a)-(i), 50 μm in (j), and 20 μm in (k), respectively.

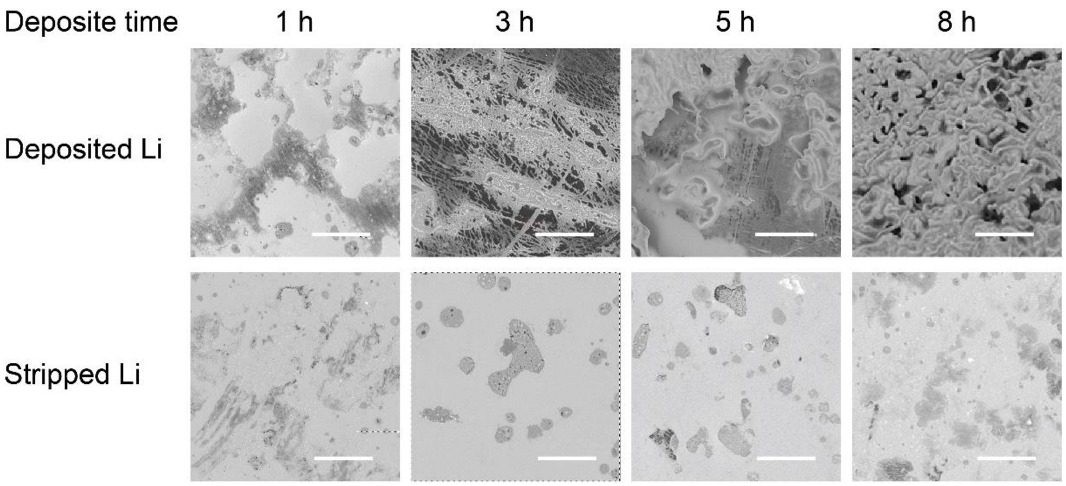

Figure S22. SEM images of lithium deposition/stripping morphology with the PE separator in Li symmetric batteries after 1, 3, 5 and 8 h. The current density is 1.5 mA cm^{-2}. The scale bar in the figure is 80 μm.

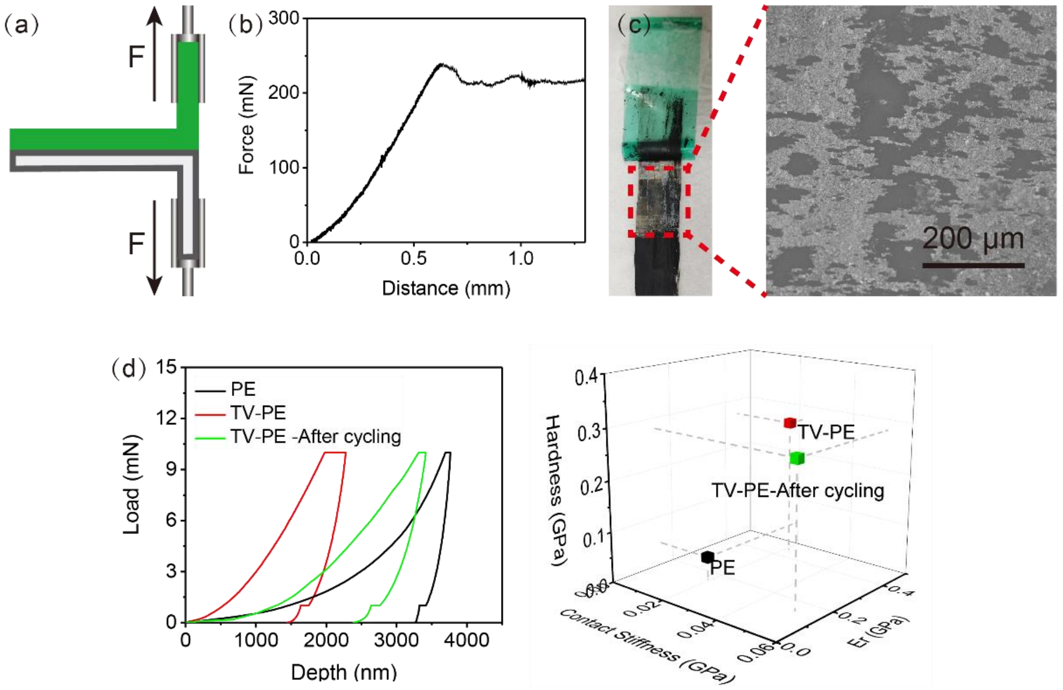

Figure S23. 180° peeling and indentation test of the TV layer and separators. (a) 180° peeling test model. (b) Peeling force and the displacement distance curve of the TV-layer. (c) The optical photograph and the micro-morphology of the peeled TV-PE separator. (d) Nano-indentation and depth curve of the pristine PE, TV-PE before and after cycling. (e) Contact stiffness, hardness and the indentation modulus of the pristine PE, TV-PE before and after cycling.

According to **Figure S23**b, the max peeling force of 239.7 mN corresponds to the displacement distance of 0.63 mm and the width of 10 mm of the separator sample. The peeling strength of the TV layer is calculated as following:

$$\frac{239.7\ mN}{0.63\ mm \times 10mm} = 38\ kPa$$

$$\frac{239.7\ mN}{0.63\ mm} = 0.38\ kN\ m^{-1} = 3.9\ gf\ mm^{-1}$$

The adhesive force of the coating on PE matrix was tested by the 180° peeling test. As **Fig. S23**a-**S23**b shown that TV-layer has a medium binding strength of 3.8 kN m^{-1} equals to 3.9 gf mm^{-1}, which is comparable to the Al_2O_3 (7 gf mm^{-1}) and the $Mg(OH)_2$ (4 gf mm^{-1}) functional layer [4].

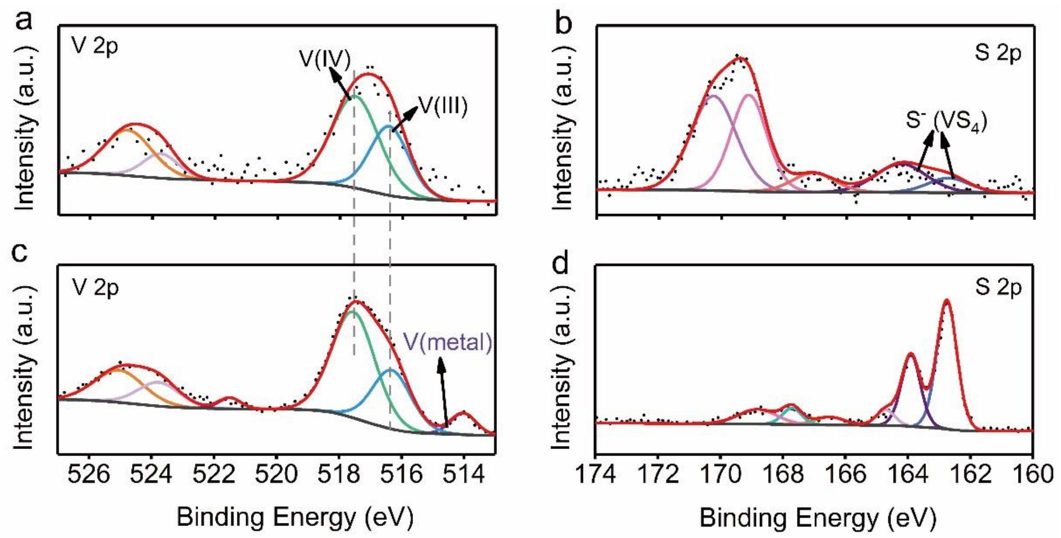

Figure S24. XPS results of the TV layer (a-b) before and (c-d) after cycling in a Li symmetric battery.

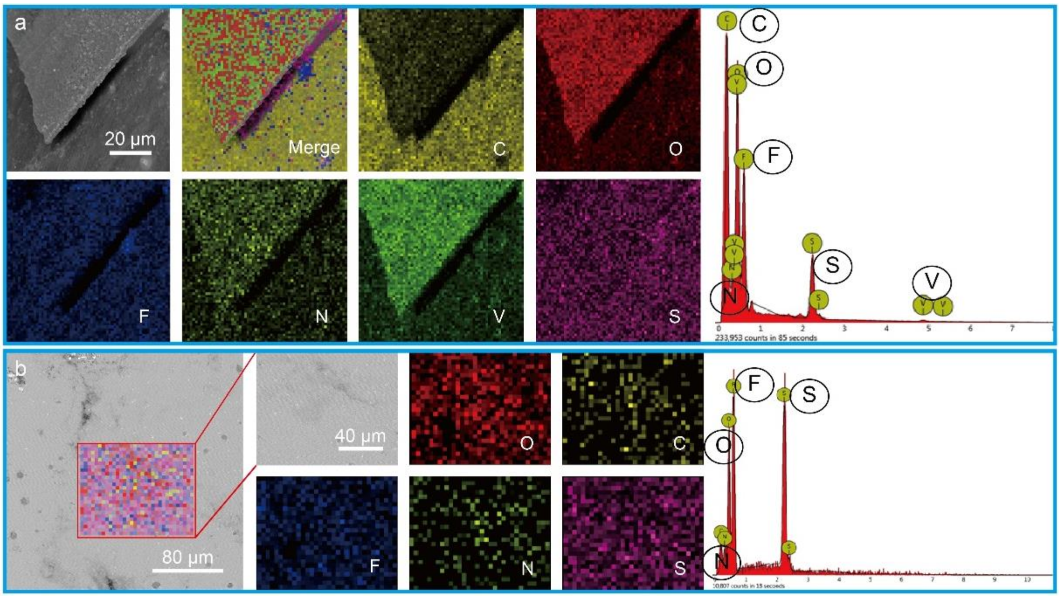

Figure S25. SEM images and the corresponding EDS mapping of the cycled TV-PE separator and the cycled lithium surface in the symmetric lithium battery with the TV-PE separator.

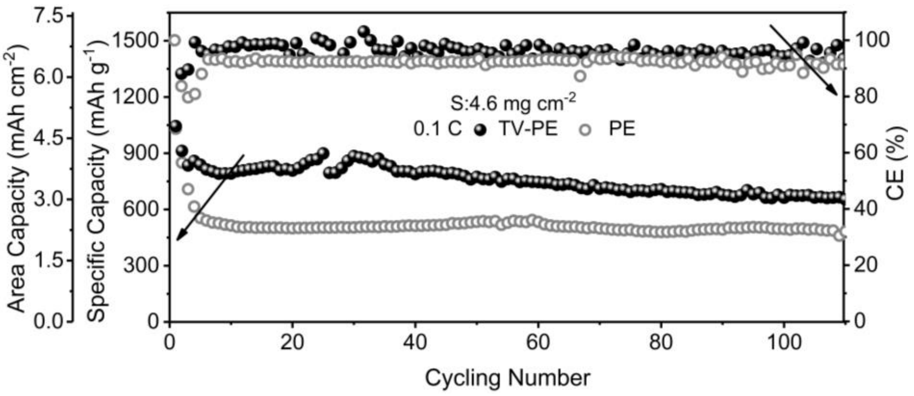

Figure S26. Cycling performance and corresponding Coulombic efficiencies of LSBs with TV-PE and PE separator at 0.1 C.

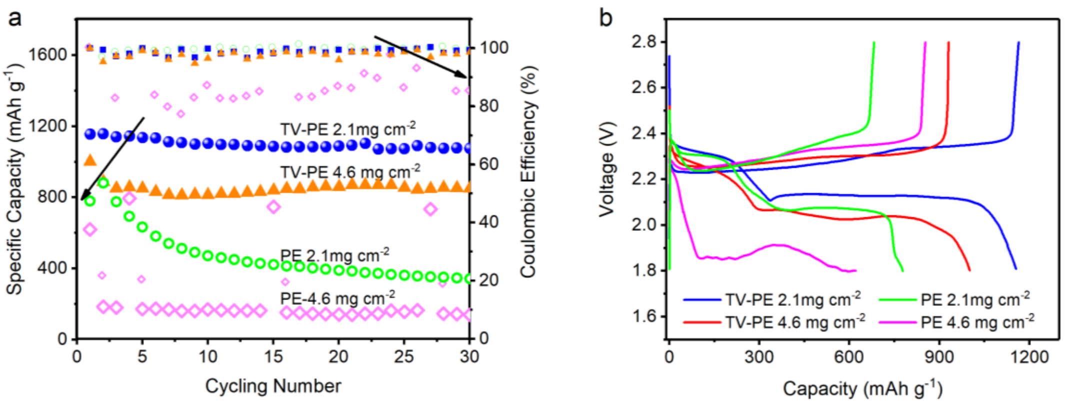

Figure S27. (a) Cycling performance and the corresponding Coulombic efficiencies of LSBs with sulfur loading of 2.1 mg cm^{-2} and 4.6 mg cm^{-2} using different separators. The LSBs with 2.1 mg cm^{-2} sulfur loading were cycled at 0.2 C. The LSBs with 4.6 mg cm^{-2} sulfur loading were charged at 0.2 C and discharged at 0.1 C. (b) Voltage profiles of the LSBs in (a).

Noted that the LSB in **Figure 6**a-**6**b shows a relative larger polarization, lower CE than regular LSB and obvious activation phenomenon during cycling because of the high sulfur loading and the low E/S ratio. These problems can be solved by properly adjusting the discharge and charge manner or reducing the sulfur loading. As shown in **Fig. S27**, the above two methods can narrow the voltage gap and improve the CE effectively in the LSBs with TV-PE separator. In contrast, the LSBs with pristine PE separator can hardly cycle stably with high sulfur loading and decay fast during cycling with low sulfur loading. These strong comparisons also highlight the electrochemistry regulation of the designed TV layer in both the lithium anode and sulfur cathode.

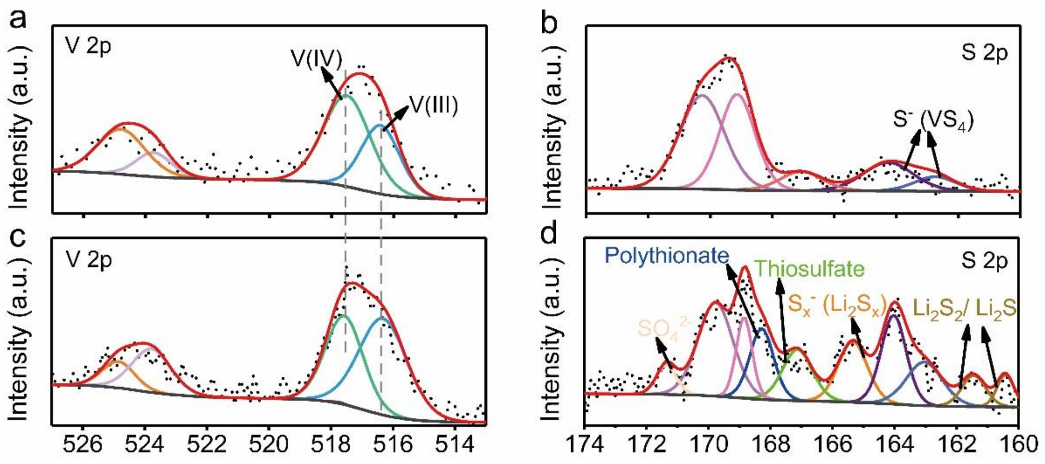

Figure S28. XPS results of the TV layer (a-b) before and (c-d) after interacting with LiPSs solution.

After interacting with polysulfides, the TV layer was rinsed with DOL and DME for several times and dried in the glove box for further characterization of the chemical interactions between TV layer and polysulfides by XPS, as displayed in **Figure S28**.

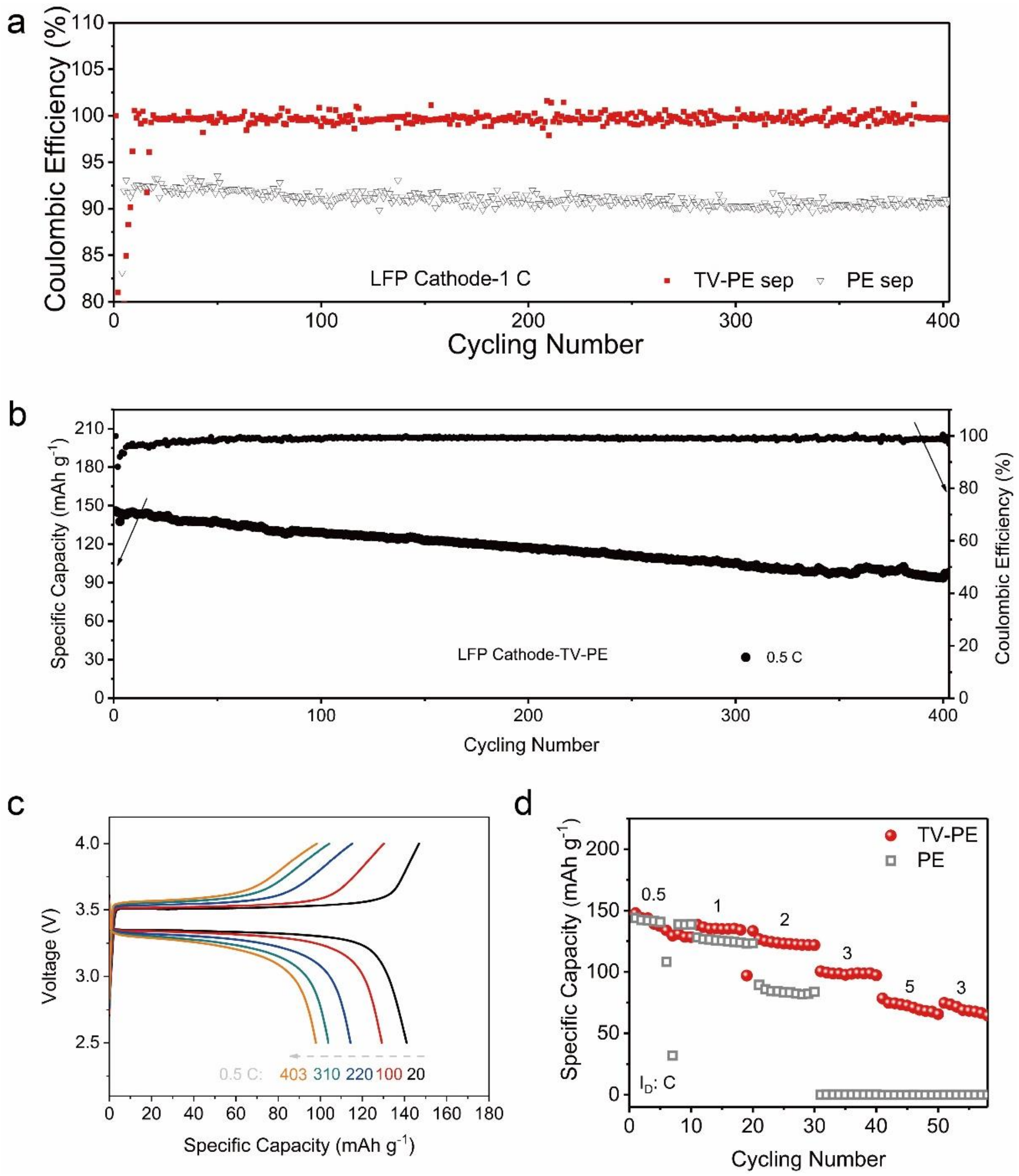

Figure S29. (a) Comparation of the CE of the LMB- LFP batteries with TV-PE and PE separator. Long cycling performance (b) and voltage profile (c) of the LMB-LFP batteries with TV-PE separator at 0.5 C. (d) Comparation of the rate performance of commercial LFP cathode coupled with Li metal anode using TV-PE and PE separator.

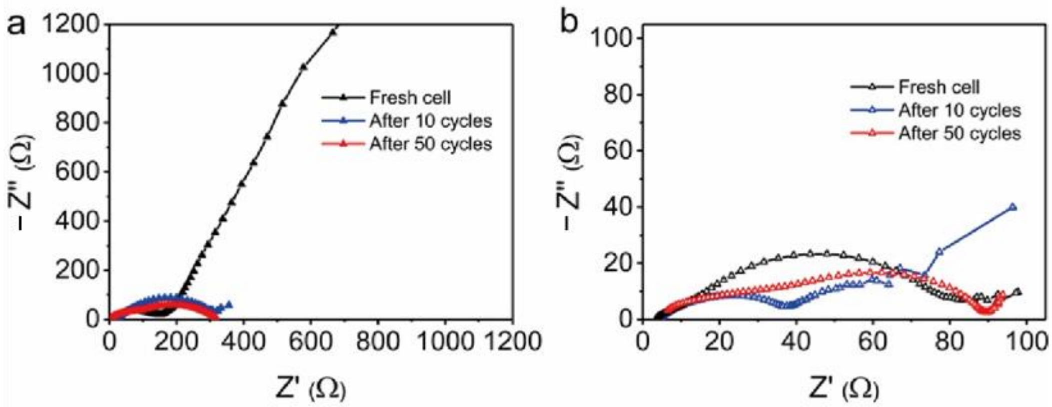

Figure S30. EIS results of LMNCO- full cells before and after cycling using (a) TV-PE and (b) TV-PE separator with single-side coating.

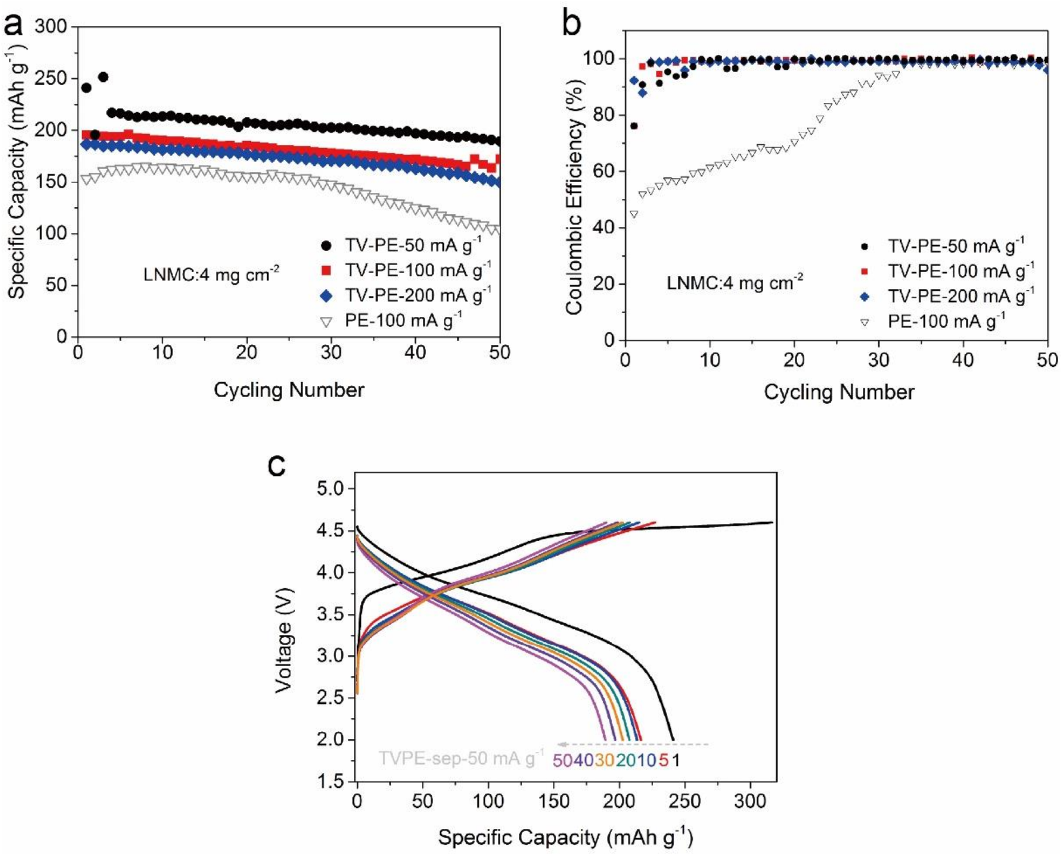

Figure S31. The electrochemical performance of the Li-rich cathode batteries at low current densities with TV-PE separator and PE separator. (a) The cycling performance, (b) the Coulombic efficiencies in (a), (c) the voltage profiles of different cycles at 50 mA g^{-1}.

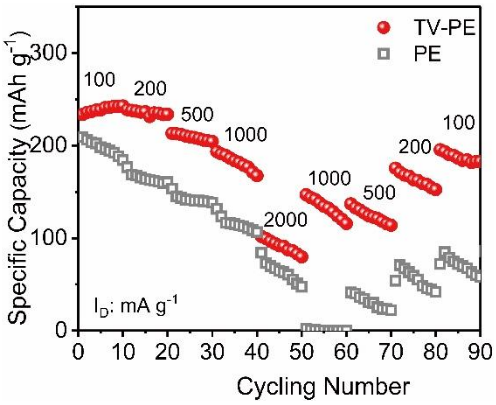

Figure S32. Comparation of the rate performances of LMBs-LMNC-622 coupled with TV-PE and PE separators.

LFP/Li battery and LMNC-622/Li battery coupled with the TV-PE separator show a better rate capability than that with PE separator. Higher capacities are delivered with the TV-PE separator, especially at the high current densities, which is attributed to its better Li ion regulation.

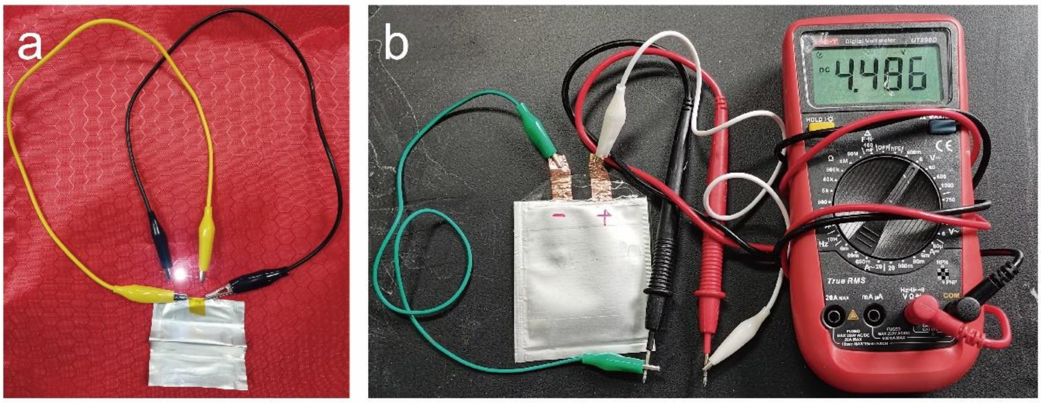

Figure S33. (a) The white LED luminescence demonstration of the soft-packaged thin LMB with LMNC-622 cathode. (b) The open-circuit voltage test of the soft-packaged thin LMB.

Table S3 Comparison of the Lithium deposition performance through the separator modification strategy in some published papers and our TV-polyolefin separator.

Strategy	Layer thickness (mass loading)	Li symmetric cells: Cycling life Current density/Area capacity	Electrolyte	**Refs.**
TV-PP separator	1.5 μm	**6000 h** 1 mA cm^{-2}/1 mAh cm^{-2}	1 M LiTFSI in DME/DOL with 2 wt% $LiNO_3$	**This work**
TV-PE separator	1.5 μm 15 in total (0.15 mg cm^{-2})	**4200 h** 3.6 mA cm^{-2}/3.5 mAh cm^{-2} **4200 h** 8.2 mA cm^{-2}/3.5 mAh cm^{-2}	1 M LiTFSI in DME/DOL with 2 wt% $LiNO_3$	
		**3200 h** 1 mA cm^{-2}/3.5 mAh cm^{-2}	1 M $LiPF_6$ in EC/DMC	
PLLZ@PP separator	10 μm	**1000 h** (1 mA cm^{-2}/1 mAh cm^{-2}) 600 h (1 mA cm^{-2}/3 mA cm^{-2})	1 M LiTFSI in DME/DOL	[5]
GO-g-PAM@PP separator	>5 μm	**2600 h** 2 mA cm^{-2}	1 M LiTFSI in DME/DOL with 1 wt% $LiNO_3$	[6]
Lignosulfonate@PP separator	1.7 μm	**400 h** 1 mA cm^{-2}/1 mAh cm^{-2}	1 M LiTFSI in DME/DOL with 2 wt% $LiNO_3$	[7]
PP/$MnCO_3$ separator	~3.8 μm	**2000 h** 1 mA cm^{-2}/1 mAh cm^{-2} **2000 h** 2 mA cm^{-2}/1 mAh cm^{-2} **2000 h** 5 mA cm^{-2}/1 mAh cm^{-2}	1 M LiTFSI in DME/DOL with 1 wt% $LiNO_3$	[8]
SiO_2@Celgard 2250 separator	~1 μm	**350 h** 0.25 mA cm^{-2}/1 mAh cm^{-2} >400 h 3 mA cm^{-2}/3 mAh cm^{-2}	1.0 M $LiPF_6$ in EC/DEC with 2 wt% VC	[9]
Si-PP-Si separator	2 μm (~0.2 mg cm^{-2})	**1000 h** 0.5 mA cm^{-2}/1 mAh cm^{-2}	1 M $LiPF_6$ in EC: DEC = 1:1 wt/wt with 10 wt%	[10]

			FEC and 1 wt% VC	
UiO-66-SO_3Li/PVDF separator (22)	22 μm in total	**~1000 h**<br>0.5 mA cm^{-2}/0.25 mAh cm^{-2}<br>**~1400 h**<br>2 mA cm^{-2}/0.5 mAh cm^{-2}<br>**~1400 h**<br>5 mA cm^{-2}/0.5 mAh cm^{-2}	1 M LiTFSI in DME/DOL with 2 wt% $LiNO_3$	[11]
PZT/PP separator	4 μm	**200 h**<br>2 mA cm^{-2}/0.5 mAh cm^{-2}	1 M $LiPF_6$ in EC/DMC	[12]
CGC separator	2.5 μm-CNF layer<br>20 μm in total	**620 h**<br>0.65 mA cm^{-2}/0.65 mAh cm^{-2}	LP40 electrolyte	[13]
D-HVS@PP separator	/<br>(0.14 mg cm^{-2})	**400 h**<br>5 mA cm^{-2}/0.5 mAh cm^{-2}	/	[14]

Table S4 Comparison of the modified separator properties in some published papers and our TV-polyolefin separator.

**Layer Thickness/μm**	**Total thickness/μm**	**Layer loading/ mg cm^{-2}**	**t_{Li+}**	**Cycling Life/h**	**Refs.**
1.5	15	0.17	0.68	4300	This work
20	45	/	0.68	150	[15]
5	30	/	0.42	800	[16]
1	25	/	0.53	400	[17]
2.7	21	/	0.49	/	[18]
5	23	2.4	0.454	/	[19]
2	27	0.2	/	1000	[20]
4	23	0.2	/	/	[21]
10	35	/	/	1000	[22]
10	35	0.54	/	/	[23]
4	24	/	/	2000	[1c]
11.3	36.3	0.25	/	/	[24]

REFERENCE

[1] a) J. W. Meng, F. L. Chu, J. L. Hu, C. L. Li, *Adv. Funct. Mater.* **2019** , *29*, 13, 201902220*;* b) H. D. Shi, M. Yue, C. J. Zhang, Y. F. Dong, P. F. Lu, S. H. Zheng, H. J. Huang, J. Chen, P. C. Wen, Z. C. Xu, Q. Zheng, X. F. Li, Y. Yu, Z. S. Wu, *ACS Nano* **2020**, *14*, 8678; c) J. Yan, F. Q. Liu, Z. Y. Hu, J. Gao, W. D. Zhou, H. Huo, J. J. Zhou, L. Li, *Nano Lett.* **2020**, *20*, 3798.

[2] X. Y. Zhang, A. X. Wang, X. J. Liu, J. Y. Luo, *Accounts Chem. Res.* **2019**, *52*, 3223.

[3] a) D. Aurbach, M. L. Daroux, P. W. Faguy, E. Yeager, *J. Electrochem. Soc.* **1987**, *134*, 1611; b) D. M. Kang, N. Hart, J. Koh, L. G. Ma, W. B. Liang, J. Xu, S. Sardar, J. P. Lemmon, *Energy Storage Mater.* **2020**, *24*, 618.

[4] B. Jung, B. Lee, Y. C. Jeong, J. Lee, S. R. Yang, H. Kim, M. Park, *J. Power Sources* **2019**, *427*, 271.

[5] H. Huo, X. Li, Y. Chen, J. Liang, S. Deng, X. Gao, K. Doyle-Davis, R. Li, X. Guo, Y.

Shen, C.-W. Nan, X. Sun, *Energy Storage Mater.* **2020**, *29*, 361.
[6] C. Li, S. Liu, C. Shi, G. Liang, Z. Lu, R. Fu, D. Wu, *Nat. Commun.* **2019**, *10*, 1363.
[7] J. Liu, R. Xu, C. Yan, H. Yuan, J.-F. Ding, Y. Xiao, T.-Q. Yuan, J.-Q. Huang, *Energy Storage Mater.* **2020**, *30*, 27.
[8] J. Yan, F. Liu, Z. Hu, J. Gao, W. Zhou, H. Huo, J. Zhou, L. Li, *Nano Lett.* **2020**, *20*, 3798.
[9] J. Liang, Q. Chen, X. Liao, P. Yao, B. Zhu, G. Lv, X. Wang, X. Chen, J. Zhu, *Angew. Chem., Int. Ed.* **2020**, *59*, 6561.
[10] X. Chen, R. Zhang, R. Zhao, X. Qi, K. Li, Q. Sun, M. Ma, L. Qie, Y. Huang, *Energy Storage Mater.* **2020**, *31*, 181.
[11] Z. Q. Wang, W. Y. Huang, J. C. Hua, Y. D. Wang, H. C. Yi, W. G. Zhao, Q. H. Zhao, H. Jia, B. Fei, F. Pan, *Small Methods* **2020**, *4*, 9.
[12] Z. Hu, F. Liu, J. Gao, W. Zhou, H. Huo, J. Zhou, L. Li, *Adv. Funct. Mater.* **2019**, *30*, 201907020.
[13] R. Pan, R. Sun, Z. Wang, J. Lindh, K. Edström, M. Strømme, L. Nyholm, *Nano Energy* **2019**, *55*, 316.
[14] J. Wang, S. Yi, J. Liu, S. Sun, Y. Liu, D. Yang, K. Xi, G. Gao, A. Abdelkader, W. Yan, S. Ding, R. V. Kumar, *ACS Nano* **2020**, *14*, 9819.
[15] Z. D. Hao, Y. Wu, Q. Zhao, J. D. Tang, Q. Q. Zhang, X. X. Ke, J. B. Liu, Y. H. Jin, H. Wang, *Adv. Funct. Mater.* **2021**, *31*, 9, 202102938.
[16] D. H. Han, M. Zhang, P. X. Lu, Y. L. Wan, Q. L. Chen, H. Y. Niu, Z. W. Yu, *J. Energy Chem.* **2021**, *52*, 75.
[17] Y. J. Li, S. Y. Lin, D. D. Wang, T. T. Gao, J. W. Song, P. Zhou, Z. K. Xu, Z. H. Yang, N. Xiao, S. J. Guo, *Adv. Mater.* **2020**, *32*, 10, 201906722.
[18] H. Zhang, L. Sheng, Y. Z. Bai, S. J. Song, G. J. Liu, H. R. Xue, T. Wang, X. L. Huang, J. P. He, *Adv. Eng. Mater.* **2020**, *22*, 7, 201901545.
[19] T. Yim, S. H. Han, N. H. Park, M. S. Park, J. H. Lee, J. Shin, J. W. Choi, Y. Jung, Y. N. Jo, J. S. Yu, K. J. Kim, *Adv. Funct. Mater.* **2016**, *26*, 201602498.
[20] X. Chen, R. Y. Zhang, R. R. Zhao, X. Q. Qi, K. J. Li, Q. Sun, M. Y. Ma, L. Qie, Y. H. Huang, *Energy Storage Mater.* **2020**, *31*, 181.

[21] Z. Q. Ye, Y. Jiang, T. Feng, Z. H. Wang, L. Li, F. Wu, R. J. Chen, *Nano Energy* **2020**, *70*, 9.

[22] H. Y. Huo, X. N. Li, Y. Chen, J. N. Liang, S. X. Deng, X. J. Gao, K. Doyle-Davis, R. Y. Li, X. X. Guo, Y. Shen, C. W. Nan, X. L. Sun, *Energy Storage Mater.* **2020**, *29*, 361.

[23] P. Y. Zhai, H. J. Peng, X. B. Cheng, L. Zhu, J. Q. Huang, W. C. Zhu, Q. Zhang, *Energy Storage Mater.* **2017**, *7*, 56.

[24] D. L. Fang, Y. L. Wang, X. Z. Liu, J. Yu, C. Qian, S. M. Chen, X. Wang, S. J. Zhang, *ACS Nano* **2019**, *13*, 1563.
